# Comprehensive Survey of Using Machine Learning in the COVID-19 Pandemic

**DOI:** 10.3390/diagnostics11071155

**Published:** 2021-06-24

**Authors:** Nora El-Rashidy, Samir Abdelrazik, Tamer Abuhmed, Eslam Amer, Farman Ali, Jong-Wan Hu, Shaker El-Sappagh

**Affiliations:** 1 Machine Learning and Information Retrieval Department, Faculty of Artificial Intelligence, Kafrelsheiksh University, Kafrelsheiksh 13518, Egypt; 2 Information System Department, Faculty of Computer Science and Information Systems, Mansoura University, Mansoura 13518, Egypt; samir.abdelrazek@mans.edu.eg; 3 College of Computing and Informatics, Sungkyunkwan University, Seoul 03063, Korea; 4 Faculty of Computer Science, Misr International University, Cairo 11828, Egypt; eslam.amer@miuegypt.edu.eg; 5 Department of Software, Sejong University, Seoul 05006, Korea; farmankanju@sejong.ac.kr; 6 Department of Civil and Environmental Engineering, Incheon National University, Incheon 22012, Korea; 7 Centro Singular de Investigación en Tecnoloxías Intelixentes (CiTIUS), Universidade de Santiago de Compostela, 15782 Santiago de Compostela, Spain; 8 Information Systems Department, Faculty of Computers and Artificial Intelligence, Benha University, Banha 13518, Egypt

**Keywords:** artificial intelligence, deep learning, COVID_19

## Abstract

Since December 2019, the global health population has faced the rapid spreading of coronavirus disease (COVID-19). With the incremental acceleration of the number of infected cases, the World Health Organization (WHO) has reported COVID-19 as an epidemic that puts a heavy burden on healthcare sectors in almost every country. The potential of artificial intelligence (AI) in this context is difficult to ignore. AI companies have been racing to develop innovative tools that contribute to arm the world against this pandemic and minimize the disruption that it may cause. The main objective of this study is to survey the decisive role of AI as a technology used to fight against the COVID-19 pandemic. Five significant applications of AI for COVID-19 were found, including (1) COVID-19 diagnosis using various data types (e.g., images, sound, and text); (2) estimation of the possible future spread of the disease based on the current confirmed cases; (3) association between COVID-19 infection and patient characteristics; (4) vaccine development and drug interaction; and (5) development of supporting applications. This study also introduces a comparison between current COVID-19 datasets. Based on the limitations of the current literature, this review highlights the open research challenges that could inspire the future application of AI in COVID-19.

## 1. Introduction

The first coronavirus was detected among humans in 1960 and was known as a human coronavirus (HCoV) [1]. It caused mild diseases to the lower and upper respiratory that led to acute respiratory failure in some cases [2]. The situation became more serious in 2003 with the appearance of a severe acute respiratory syndrome (SARS-CoV) in China [3]. At that time, nearly 1 million people were affected by SARS-COV, with a mortality rate of 9.5%. The spread of this virus stopped by isolating the infected people and detecting the causes of infections. Subsequent experiments in wild animals have shown that SARS-COV exists in cats and bats [4]. Therefore, it was believed that the virus spread to humans from bats and cats, then spreading from human to human [5]. The situation has remained stable from 2004 till the appearance of another dangerous virus in 2102, known as the Middle East respiratory syndrome coronavirus (MERS-CoV) [6]. The MERS-CoV appeared firstly in patients with acute pneumonia in the Kingdom of Saudi Arabia (KSA) [7,8]. Although MERS-CoV has a lower spread rate than SARS, the rate of mortality among MERS-CoV patients was higher [9]. By the end of 2018, about 2500 MERS-CoV was reported with a mortality rate up to 30%.

In 2019, the world faced another coronavirus that started to spread in Wuhan, China, known as SARS-COV-2, which causes COVID-19. COVID-19 is a rapidly spreading disease that transmits through contact with the infected person. Respiratory droplets (direct contact) and aerosolized droplets (indirect contact) are considered the main cause of infections [10,11]. If there is no vaccine for COVID-19 available, non-pharmaceutical interventions such as personal hygiene and social distancing are the most precautionary measure against COVID-19 outbreak [12]. It is noteworthy to understand that at its peak, the pandemic overloads existing medical centers. Thus, emergency and the intensive care centers have expanded beyond their capacity to serve the increasing number of infected subjects. COVID-19 usually starts with mild symptoms, such as fever and cough, and changes gradually, causing organ failure and death [13]. Therefore, in such pandemics, the medical expertise and even the patient’s relatives need to make fast and educated decisions to reduce the sudden deterioration.

The main challenges of COVID-19 are its identification and classification. This is due to its interaction with other lung infections. Currently, reverse transcriptase quantitative polymerase chain reaction (RT-qPCR) is the standard for COVID-19 identification [14]. Small quantities of viral RNA are collected from the nasal swab and then amplified to be identified with virus detection techniques. Unfortunately, the traditional way for RT-qPCR is time-consuming and requires the involvement of medical expertise, which may not be available. On the other hand, some studies have shown high false-positive rates for RT-qPCR testing [15,16,17]. Therefore, virology, medical, and artificial intelligence (AI) scientists have stood right up to limit this crisis with innovative approaches.

In this regard, the AI community provided significant solutions that could help detect, predict, and treat COVID-19 [18]. Textual and radiation data are considered basic data types for a COVID-19 diagnosis. Textual data include patient records, PCR analysis, mobility data, etc. Radiation data include chest CT, X-ray, etc. AI has been commonly used to solve several problems based on various data types (i.e., text, image, video, signals, etc.). Machine learning (ML) algorithms utilize the available data to learn and adapt models to solve specific tasks. The main contribution of this paper is to survey the state of the art in AI applications in a COVID-19 context, from different perspectives and various disciplines.

- We discuss the detailed characteristics of COVID-19 symptoms, behaviors, and patterns.
- We investigate the role of automated analysis and diagnosis of COVID-19 based on the WHO statistics worldwide.
- We propose a taxonomy for using AI, big data, and statistics in COVID-19 diagnosis, prediction, and treatment. Based on this taxonomy, a comprehensive survey of current AI literature is provided.
- We collect the details about all available COVID-19 datasets (i.e., textual data, medical images, and speech data).
- We explore the limitations of the current literature of AI applications in the COVID-19 domain and draw the directions for future improvements that could handle these challenges.

The rest of the article is organized as follows. Section 2 introduces the taxonomy of using AI for classifying COVID-19. Section 3 presents a survey of the literature for using AI in a COVID-19 context. Section 3 shows a comparison among COVID-19 datasets. Section 4 is a discussion of the results discovered from studying the literature. Limitations of the current solutions and future directions are introduced in Section 5, and the paper is concluded in Section 6. Table 1 include all terms and its abbreviation.

## 2. The Study Taxonomy

**Figure 1:**
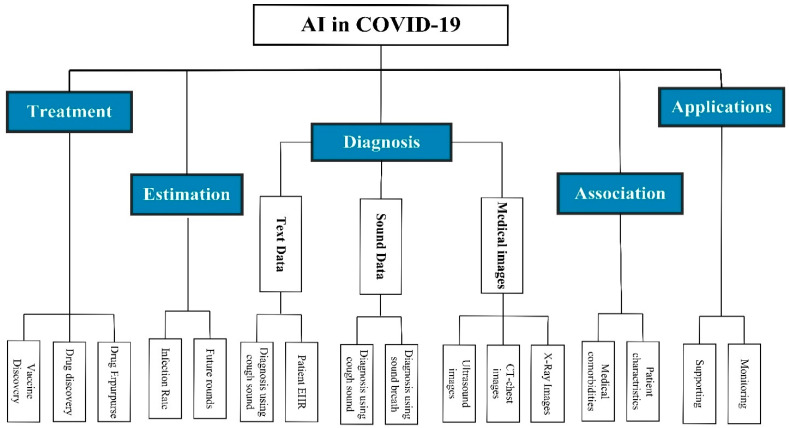
Taxonomy of using AI in COVID-19.

The world is facing the COVID-19 pandemic, and it needs to be managed. A reliable estimation of future confirmed cases, identification of disease pathology, and an effective vaccine to slow down the spread of infection are highly needed. This section presents a taxonomy that summarized the role of AI and information communication technology (ICT) in facing the COVID-19 pandemic. As shown in Figure 1, this taxonomy divides the literature into five main research domains: (1) Diagnosis, utilizing ML and DL in COVID-19 diagnosis based on various types of data, such as medical imagery data (CT chest scan, X-ray images, and ultrasound images), respiratory data (breathing and cough sound), or other data (e.g., symptoms); (2) Estimation, providing statistical estimation about the expected future rounds and infection rate; (3) Association, using ML and DL techniques to examine the correlation between the risk of COVID-19 infection and other patients’ data (e.g., patient’s characteristics and medical comorbidities); (4) Treatment, by developing models that help to analyze the virus protein and RNA sequences in a way that helps in drug repurposing and drug development; (5) Application, by developing supportive tools that help in taking preemptive actions, such as intelligent chatbots, monitoring systems, and supportive robotics. Figure 1 shows the taxonomy of using AI in COVID-19 management.

### 2.1. COVID-19 Diagnosis

#### 2.1.1. Diagnosis Using Medical Images

Although medical images, such as those from CT scans and X-rays, could provide valuable pathological information, only the qualitative assessment is written in the radiological report. This is due to the lack of computerized tools that measure the infected areas and their changes. Therefore, the changes across the medical images are often ignored. On the other hand, contouring the infected areas in the CT scan is recommended for quantitative evaluation. Unfortunately, manual contouring is time-consuming, tedious, and may lead to discrepancies in the assessment. With this in mind, fast and automated contouring tools for COVID-19 medical images are an urgent need to face the fast-growing COVID-19 pandemic. The following subsections survey the ML and DL models used to make auto controlling, segmentation, and classification of COVID-19 medical images for disease diagnosis.

##### Diagnosis Using CT Chest Scans

Several studies have developed DL models for COVID-19 identification and diagnosis, with promising results, which are mainly based on CT chest images [19]. For example, [20] proposed a DL model to extract the visual features from CT chest scan images. The study used the extracted features to differentiate between COVID-19 and other pneumonia diseases. However, the proposed system was not able to define the progression of COVID-19 disease. Ahuja et al. [21] developed a CNN model to analyze and detect COVID-19. The developed model depended on extracting and specifying opacities in the lung images, and it achieved 92.21% and 98.50% for sensitivity and specificity, respectively. The developed system is considered robust in terms of pixel spacing. Jaiswal et al. [22] provided a DL model for CT segmentation and detection of COVID-19 infection. Xue et al. [23] did a similar task by developing a classification model to discriminate COVID-19 and other non-pneumonia, with an accuracy of 86.30%. In [24], Ozturk et al. proposed a 3D CNN model to classify COVID-19 patients from normal ones using chest CT images and other images of viral pneumonia. First, the infected regions were segmented from a CT chest scan using the 3D CNN model. Then, these separated images were categorized using the location attention model. Finally, the noisy-OR Bayesian function has been used to calculate the confidence score.

Due to the limited access to COVID-19 datasets, several studies reported that the pre-trained model and transfer learning became the most effective techniques to build diagnosis and prediction models for COVID-19 [25,26,27]. For example, Jaiswal et al. [22] utilized deep transfer learning to build a classification model for chest CT scans using the DenseNet201 pre-trained model. A total of 1260 CT images for COVID-19 patients and 1232 CT chest images for health patients were used to train and test the DenseNet201 model. The proposed model achieved promising results in terms of various metrics, including precision, recall, F-measure, and accuracy, at 96.20%, 96.20%, 96.20%, and 96.21%, respectively. In [28], Pathak et al. also used transfer learning and the ResNet50 pre-trained model to build a 2D classification model for COVID-19 to classify infected CT chest images from the normal images. The proposed model achieved a training accuracy of 96.32% and a testing accuracy of 93.11%. However, the model takes a long training time. Wang et al. [29] proposed a segmentation and classification model for the CT scans, the pipeline was divided into two main steps. First, the segmentation step was based on DL models (i.e., U-Net, 3D U-Net++, and V-Net). Second, the classification was by using a pre-trained model (i.e., ResNet-50, and DPN-92). The model was evaluated using CT chest scans of 732 cases and resulted in a classification model with an AUC = 99.01%. In [30], Weng et al. developed a model that analyzed the changes in the CT chest images of the infected patients. They developed a CNN model that utilized an inception pre-trained model and transfers the learning technique to build an effective model for diagnosis. This model achieved a performance of 89.66 % for accuracy and saved time.

Other studies tried to overcome the shortage in the CT datasets by training the model in various types of pneumonia. For example, in [31], Cheng et al. proposed a multiclass deep CNN model. The system evaluated more than ten thousand CT chest images from four categories, including influenza, non-viral pneumonia, COVID-19, and non-pneumonia subjects. The proposed system was evaluated based on 1940 samples, with an AUC, sensitivity, and specificity of 95.76%, 90.10%, and 97.16 %, respectively. The same procedure has been followed by [26,28,32,33,34,35,36].

In [37], Farid et al. proposed a prediction model that predicted the recurrences in both COVID-19 and SARS cases. They composited a hyper feature extraction technique of the main four filters, namely, a Gabor filter, MPEG-7 histogram filter, fuzzy-64, and local binary histogram. Then, they built a hybrid classification technique of CNN and ML models to achieve a high accuracy in prediction. The proposed model enhanced the performance and reduced the false-positive rate after applying feature optimization techniques. The model was evaluated by using only 51 images extracted from the Kaggle benchmark dataset. As it is clearly noticed, the evaluation of the model using such a small dataset does not guarantee the generalization ability.

Some studies tried to examine the relationship between CT scans and symptoms. For example, brahmin et al. [38] analyzed 121 CT chest images of positive COVID-19 cases. They found that the prevalence of symptoms and the signs of diseases increased with time from the onset time. In [39], Xueyan et al. proposed a COVID-19 prediction system that integrated CT chest scan images, patient demographics (e.g., age, weight, and sex), clinical symptoms (e.g., fever, cough, and sputum), and laboratory test (e.g., WBC, lymphocytes, neutrophils, etc.). The authors reported that the presence of patient symptoms and laboratory tests gave the classification model a better performance—84.34% for sensitivity ($confidence\;interval\;\left[CI\right]=77.1\%,\;90.0\%\;;\;p=0.662)$—compared to a CNN model that used CT chest images only, which achieved a sensitivity of 82.6% $\left(CI=76.2\%,\;89.4\%\;;\;p=1\right)$. The *p*-value clarifies the significant difference with respect to the integrated model. Table 2 lists more COVID-19 classification models based on CT-scan images.

Chest CT scan-based detection of COVID-19 is considered difficult, as patients need to be moved to the CT room with a danger of radiation, and machines need a high level of cleaning after each use. Therefore, a CT chest scan is not recommended as the main identification tool for COVID-19.

From the previous table (Table 2), we could notice the following: (1) 60% of the studies build binary classification models, 34% built multiclass classification models, and 6% used object detection techniques to detect COVID-19; (2) 48% used transfer learning to overcome the shortage in data; (3) studies that build binary classification models achieve better results than binary-class classification models; and (4) 66% of the studies used DL models for COVID-19 classification, whereas 34% used conventional ML models (i.e., SVM, RF, DT, etc.). The best results are achieved when using pretrained models with the GAN network model and ResNet pretrained model [47]. This is due to using a pretrained model to fine-tune the network parameter and use GAN to provide a robust model and overcome the overfitting problem.

##### Diagnosis Using X-ray

COVID-19 radiological analysis is a common and cost-effective technique for COVID-19 detection, especially in the intermediate stage of the disease. Medical experts in [48] reported that X-ray of COVID-19 patients presented no change in the early stages of the disease. However, with disease progression, two main observations are commonly observed in X-ray images, including patchy infiltrates in the lower and upper zones of the lungs. Moreover, the transfer of digital X-ray images does not need any transportation from the point of acquisition to the point of analysis, making the diagnostic operation extremely fast. Moreover, the portable X-ray machines allow testing within an isolation ward. These machines minimize the main need for additional personal protective equipment. It also minimizes the risk of hospital-acquired infections for patients. Therefore, several recent studies utilized X-ray in COVID-19 diagnosis. The main goal of this subsection is to discuss the state of the art of COVID-19 diagnosis and detection based on X-ray images.

Several recent studies applied different ML and DL techniques in diagnosing COVID-19 based on radiographic imagery. For example, Elisha et al. [49] provided an ML model for COVID-19 diagnosis. The developed model was used to examine patients’ similarities according to the X-ray images. It was trained using 1384 COVID-19 patients with ages ranging from 18 and 63 and tested using 350 images. The results in accuracy reached 89.7%, and the AUC reached 94.0%. Other researchers utilized a pre-trained model to improve the model performance. For example, in [50], the authors provided a diagnostic model for COVID-19 using transfer learning. Thirteen pre-trained models, such as VGG, AlexNet, ResNet, etc., were used to extract features from 380 X-ray images; then, SVM was used for the classification. Authors reported that ResNet with SVM gave the highest accuracy of 95.33% in 22 independent executions. In [51], Shi et al. provided a diagnosis model called infection size aware random forest (ISARF). This model was built based on 1685 X-ray images from COVID-19 patients and 1027 from patients with pneumonia. They used VB-net to identify the lesion size and categorized them into four main groups. Finally, RF was used to provide the final classification decisions. The model provided accuracy, specificity, and sensitivity of 87.9%, 83.3%, and 90.6%, respectively. Kiran et al. [52] presented a multi-image augmented model using the CNN model. This model enhanced the COVID-19 detection process based on chest X-ray and chest CT scan images. The main objective of this study was to provide medical experts with a more accurate diagnosis system as the integration of X-rays and CT scans will ease the detection process of finding changes in human lungs with zero false-positive and false-negative rates. The model was trained on 19 COVID-19 cases and 50 cases of non-COVID-19. The classification accuracy reached 99.44% for X-ray and 95.38% for classifying CT scan images. Kevser and Ferhat [53] presented a DL transfer learning technique for detecting COVID-19 based on chest X-ray images. The authors utilized various pre-trained models, such as VGG19, VGG16, ResNet, DenseNet, and InceptionV3. They reported that using the VGG16 technique gave the highest classification accuracy of 80% among the other four proposed models. The same procedure has been followed in [35,54]. The authors in [54] used five pre-trained models, including ResNet50, InceptionResNetV2, and Xception. These models were trained on 5857 chest X-rays and 767 chest CT images. Results in classification accuracy were 84% for X-ray and 75% for CT scan. Table 3 lists more classification models based on the X-ray images.

From the previous table, we could notice the following: (1) 33% of the studies built binary classification models, 46% built Multiclassification model, and 8% used anomaly detection for COVID-19 classification (2) 68% used transfer learning to fine-tune the network parameter for a limited size dataset. (3) ML models were used in 46% of the studies, where 44% used DL models. Using feature optimization techniques with DL models enhances the detection and the classification process [64]. (4) Using data augmentation increases the size of the available dataset and therefore enhances the classification accuracy [52,65]. Using both X-rays and CT scans increase the performance of the classification model. The best performance is obtained when using object detection with a pretrained darknet model [24].

##### Diagnosis Using Ultrasound

Ultrasound (US) identification is an indoor positioning system (IPS) that is utilized to automatically detect and define the location of objects in real time with high accuracy. It is done by attaching nodes to the surface of persons, issues, and things; then, it transmits an ultrasound signal to connect their locations to microphone sensors [66]. Ultrasound is already used for various lung diseases, such as pneumonia and lung cancer [67]. The authors in [68] presented a survey study about the ultrasound findings from many types of research studies. It has been suggested as an effective method for diagnosis, especially in low-income countries with limited resources. Therefore, US has started to be the first-line examination instead of X-ray for COVID-19. However, the literature on the applicability of US in COVID-19 diagnosis is still limited. For example, the proposed approach in [69] utilized lung US to define suspected COVID-19 patients. The essential goal relied on the investigation of identifying COVID-19 during the initial outbreak. The outcome resulted in 41% of patients being COVID-19 positive, and includes 67% of them that were diagnosed with CP. They achieved 95%, 61%, and 90% in terms of accuracy, specificity, and sensitivity, respectively. In [70], the authors used 2,392,963 frames extracted from 64 videos. These videos were aggregated with three different categories (COVID-19, healthy, and pneumonia). The VGG-16 pre-trained model was used, followed by hidden layers (dense, dropout, batch normalization, and an output layer with SoftMax activation function) to identify COVID-19. The study resulted in a classification model with an accuracy of 89% and sensitivity of 96%. Lung US is also used to specify the duration of symptoms. In [71], authors used data from 28 patients (14 male and 14 female) that had a positive COVID-19 infection to investigate the utilization of US in specifying symptoms duration and disease severity. They reported that a thickness in the pleural line was observed in most patients with a long duration of the disease than those with a lesser disease duration. Pulmonary consolidation is also commonly observed in critical-case patients compared to moderate-case patients. One of the main challenges in using US in COVID-19 diagnosis is the quality of the US frames. This is due to the low penetration of the sound waves, which may result in noisy and low-resolution frames. This limitation motivated researchers to develop techniques that help in improving the quality of US images, such as noise filtering wavelet deconvolution [72] and contrast-limited histogram equalization (CLAHE) [73]. More classification models based on US images are listed in Table 4.

We could notice the following: (1) only a few studies utilized ultrasound for COVID-19 detection from the previous table; (2) 80% of the studies used DL and pretrained models to classify the images; (3) studies extracted image frames from ultrasound videos; and (4) the best performance was obtained when using the pretrained model VGG followed by hidden layers trained on a large number of frames [70].

#### 2.1.2. Diagnosis Using Respiratory Data

Respiratory data in conjunction with ML and DL could help in detecting and diagnosing COVID-19 through three main approaches [75,76]: (1) using cough sounds to classify positive and negative COVID-19 cases; (2) screening COVID-19 patients using breathing sounds and breathing rates; and (3) using patient sound to detect COVID-19 symptoms, including stress, anxiety, fatigue, etc. These speech datasets could also be used in remote diagnosis, monitoring, and screening for COVID-19 patients through telemedicine applications [27,77]. Kranthi et al. [78] provided a comprehensive survey using respiratory data for COVID-19 diagnosis.

Using the cough sound in COVID-19 diagnosis was motivated by several key findings, including the following: (1) several studies have shown that cough sounds from several diseases has distinct features, which could be used to train sophisticated AI models for diagnosis and detection [36,37,38,39,40]. This finding was confirmed by the meta-analysis in this study [41]. They reported that COVID-19 sound data include unique features that could be used in COVID-19 diagnosis, which do not overlap with other respiratory infections. In [76], the authors confirmed that the chest data they aggregated through stethoscope examination were used for COVID-19 diagnosis. (2) The WHO [79] reported that coughing is a common symptom among 67.7% of COVID-19 patients and considered to be the main source of infection.

Based on these findings, recent studies explored how the cough sound is collected from patients via various devices and used these data for COVID-19 diagnosis. For example, the authors in [80] provided an early effort in creating a breathing sound dataset for COVID-19. These data include the sound of the cough, breathing, and voice. These sounds were collected using website applications to enable sound-based diagnosis for COVID-19. In [81], Dunne et al. utilized three different datasets for diagnosis, including (1) Google’s Audioset (http://archive.is/MZMRJ) (Last access date: 17 February 2021) aggregated from YouTube videos (non-COVID-19); (2) the Corswara dataset (COVID-19); and (3) data collected at Stanford University (https://github.com/virufy/covid) (Last access date: 17 February 2021). In [82], the authors developed a mobile application that analyzed the patient’s cough sound and provided COVID-19 identification within 2 min. They built a DL model based on 328 cough sounds aggregated from 150 patients using four categories (bronchitis, asthma, COVID-19, and healthy). The developed model was able to differentiate between COVID-19 cough sounds and the other sounds with an accuracy of 98%. In [83], the authors depended on cough samples aggregated over the mobile phone from 3620 COVID-19-positive cases and built an application for COVID-19 diagnosis (known as AI4COVID-19). The study explored transfer learning techniques to overcome the COVID-19 cough training data shortage. It utilized the pre-trained model of ResNet18 to build a classification model and achieved promising results (AUC = 97.0%, specificity = 94.6%, and sensitivity = 98.5%). In [76], Brown et al. reported that respiratory sounds can be used to distinguish COVID-19 respiratory sounds from normal sounds. They used a simple binary classifier and achieved an AUC of 80%. Speech recordings from COVID-19 patients have been analyzed to categorize a patient’s health status [84]. Faezipour et al. [77] depended on sound data aggregated through web and android interfaces in building breathing tests for COVID-19 diagnosis. They reported that this would be effective, especially with the rapid increase of the required disease diagnostic tests. In [76], the authors used both cough and breathing sounds to distinguish between COVID-19 and healthy sounds. They built three binary classifiers, one for classifying COVID-19-positive cases from healthy individuals, one for distinguishing COVID-19-positive cases from asthma cases, and one for classifying COVID-19-positive and healthy cases who have a cough. They achieved an AUC of 82%, 81%, and 80% for these classification tasks, respectively.

### 2.2. Estimation of Disease Spread

Since the first confirmed case in 2019, the confirmed COVID-19 cases in all world were rapidly increased, which reached 86.7 million cases, including 1.87 million deaths by January 2021. Determining the future severity of the outbreak is considered one of the main keys to plan against this pandemic [85,86]. In this subsection, we survey the studies that are concerned with analyzing the epidemic status, measure the reproduction number and exponential growth using statistical and DL models. Such studies help prepare for the potential spread and reveal the significance of strict health measures to manage the COVID-19 pandemic.

The compartmental models are the most common models that are usually used for studying the spread of pandemics [85]. In these models, the population is assigned to specific labels, such as susceptible–infected–recovered (SIR) [87], susceptible–exposed–infected–suspected again (SEIS), etc. [88]. Such models used stochastic frameworks to forecast specific measures, such as the total number of infected people, infection rate, and estimated epidemiologic parameters (i.e., reproduction number), and show how public health strategies impact the epidemic outcome. For example, in the SIR model [89], the susceptible population is assumed to be the whole population of the region minus people that were previously infected by the disease. The infection rate is a function that utilized both the number of infections and the rate of transmission to estimate the infected population in each period. The SIR model has been used in several studies to estimate the expected growth of COVID-19. For example, the authors in [90] used the SIR model to measure the effect of social distancing in reducing the spread of infection. They tested the model with different social distances to estimate the expected spread after the reopening. Another study was conducted, at the beginning of the pandemic [91], using susceptible–exposed–infected–confirmed–removed (SEIQR), which has been built upon the SIR model to estimate the growth of COVID-19 in Wuhan, China. This study reported that the lockdown in China would help limit the spread in the rest of the world. Similarly, in [91], authors reported that the travel restrictions help in reducing the infection spread from Wuhan to the rest of the world. Hazhir et al. [92] used the susceptible–exposed–infected–recovered (SEIR) model to estimate the transmission of COVID-19 in 84 different countries. This model tracked the infection transmission rate due to the travel network for each country. SEIR was also used to forecast the pandemic peak in Japan [93]. The SIR and SEIR models were used to compute the transmission rate from people to people, from animal to people, and vice versa in [94]. Another study [95] was conducted in Egypt to predict the time of the peak and study the changes in the Egyptian behavior during Ramadan based on the SIR and SEIR models. The study measured the spread of the infection. In [96], the authors used the DL model to estimate the risk of COVID-19 spreading outside China. In [97], the authors utilized the logistic growth model to estimate the time and size of the COVID-19 peak in South Korea and China.

Other studies tried to estimate the future spread based on basic and effective reproduction numbers (R0, Re) only. In epidemiology, the basic reproduction number R0 is the expected number of infected cases that are directly infected on average by one confirmed case [98], where all populations are suspected to be infected. On the other hand, the effective reproduction number (Re) is the number of infected cases in a specific time and specific environment; therefore, it is known as Rt (Rtime) [99]. In [100], Salihu et al. estimated the expected growth and reproduction rate (R0) in Africa. Africa is considered one of the most affected regions with coronavirus in the Middle East. The trade relations with China have played a major role in aggravating the risk of African countries’ exposure to infections and spread of COVID-19 in a way that is difficult to counteract, especially with their reputation for having fragile state health systems. Salihu et al. [100] analyzed the epidemic between 1 March and 12 April 2020 using the growth estimation function [101]. This estimated the exponential growth per day at 0.22 (95% CI: 0.20–0.24) and the reproduction number at 2.37 (95% CI: 2.22–2.51). In [102], authors depended on SEIR data of suspected, exposed, infected, and recovered stocks that summarized the population groups and the changes in screening, diagnosis, and contact rate to measure the expected growth. The study resulted in a reproduction number of 2.6. In [103], the authors used Markov Chain Monte Carlo (MCMC) to estimate the reproduction number and rate based on the number of confirmed cases and deaths. The estimation results are a Re of 3.36 (94% CI: 3.20–3.64). In [104], the authors studied the correlation between weather and COVID-19 spread in Indonesia. Abdallah et al. [105] tried to estimate the epidemic spread in Kuwait using stochastic modeling, and the same procedure has been done in Iraq [106,107] and Egypt [108,109].

DL models were also used to track the spread of COVID-19 virus infection in terms of time and space. First, some studies utilized the respiratory patterns to predict tachypnea as it is the first diagnostic feature that could be common among large-scale COVID-19 patients. In [110], Yunlu et al. used a bidirectional gated recurrent unit (GRU) to predict tachypnea based on smartphone data. Second, researchers used DL models to predict the risk level. In [111], Yanfang et al. introduced an AI system (known as α-satellite) to specify hierarchic geographic risk assessment at different community levels. DNN was applied to a large scale of real-time data aggregated via smartphone sensors to estimate the risk level [112]. The aggregated data were then used in the development of an effective strategy to combat the rapid increase of the pandemic. LSTM model was used to predict the pandemic trend in Canada [113]. Shawni et al. [114] used a combined technique of LSTM and GRU to measure the negative and positive of the release and death cases of COVID-19.

Despite the importance of such studies in facing the COVID-19 pandemic, the risk of underestimation is still high due to several reasons [85], including (1) the nature of the disease is insertable with other diseases, which results in a large number of populations with mild symptoms (symptoms that similar to flu or cold) not being identified, and thus some that have died due to COVID-19 infection will not be recognized; (2) the variation in the number of tests across the countries resulted in imprecise estimations; and (3) population density, interaction, and lifestyle resulted in variations in reproduction numbers. Therefore, estimation should depend not only on statistical approximation, such as R0 and Re, but also on other factors such as socioeconomic status, population behavior and awareness, and the quality of the healthcare system in each country.

### 2.3. Association of COVID-19 and Other Healthcare Factors

Currently, no biological markers have been confirmed to predict one’s susceptibility to COVID-19. However, several studies tried to analyze the correlation between the risk of COVID-19 infection and patient age, gender, blood type, and medical conditions (e.g., diabetes, cardiovascular, density, etc.) [100,101,102,103]. The following subsections discuss this topic in detail [115,116,117,118,119,120,121].

### 2.4. Patient Characteristics

#### 2.4.1. Blood Type

The susceptibility of viral infections among specific blood types has been previously studied for various diseases. For example, Hepatitis and Norwalk were confirmed to have relations with specific blood groups [122,123]. On that basis, researchers studied the relationship between blood type and COVID-19 risk of infection. In [116], the authors analyzed the relationship between ABO blood type and the risk of COVID-19 infections. ABO blood type donates the existence of antigens in erythrocytes in A and B blood types. The results showed that the group A was correlated with a higher risk of infections in contrast to other blood types. This study surveyed the blood test among 23,386 patients in Wuhan, China. Applying statistical analysis tests (i.e., Chi-squared test) ended up in a 95% confidence interval. The same results were reached in [124]. A few studies have analyzed the association between Rh (positive and negative) and COVID-19 disease [115,125,126]. In [126], the authors reported that a positive Rh is more protected against latent toxoplasmosis.

#### 2.4.2. Age

In this current pandemic, the association between patient age, risk of COVID-19 infection, and death have received much speculation. Most articles reported that older age is considered one of the main factors for infection and mortality [127]. In [128], authors analyzed the data from 20 European countries and reported that the R2 value ranged from 0.766 to 0.803 for patients above 75. Another study measured the infection rate and case fatality rate among the population [129] and observed that Italy had a higher CFR of 9.3, followed by the Netherlands with a CFR of 7.4 for patients more than 70 years old. The study concluded that there is a strong relationship between age and fatality rate among COVID-19 patients. The same conclusion was reached by [130,131]. Table 5 shows the COVID-19 statistics according to patient age [130].

#### 2.4.3. Gender

**Figure 2:**
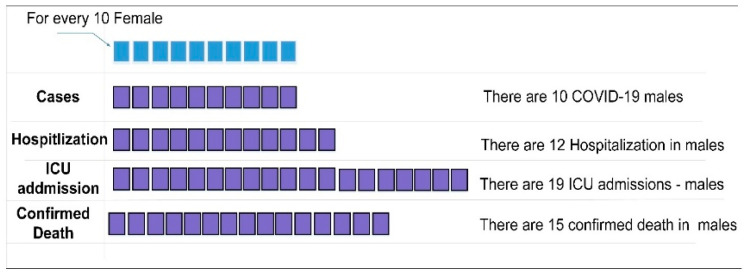
Statistics between males and females based on the number of infected cases.

The differences in men’s and women’s bodies due to their biology (sex) influence the risk of COVID-19 infection and death rate. To attribute and address these differences, several studies analyzed the infection distribution according to gender. In [132], the authors reported that there is a gender inequality among COVID-19 infections. These differences may be due to biological differences (i.e., comorbidities and immunity) or sociocultural factors (i.e., number of tests for both males and females, timelines for medical support, etc.). In [133], the authors reported that the proportion of death in males due to COVID-19 is significantly higher than in females. In [134], the authors reported that a patient’s gender might influence the risk of infection, and an immune response led to worse results in terms of infection recovery. Figure 2 shows the statistics between males and females in terms of infections, hospitalizations, admissions, and deaths. These statistics were built based on the dataset available at https://globalhealth5050.org (https://globalhealth5050.org/the-sex-gender-and-covid-19-project/dataset/, access date: 10 February 2021).

#### 2.4.4. Obesity

Obesity is an indicator of high risk among various diseases (i.e., diabetes and heart diseases) [135]. It has been associated with COVID-19 severity, admissions, and fatality rates [136]. An analytical study [137] on 16,000 COVID-19 patients conducted in the UK reported that obesity is associated with COVID-19 death with a hazard rate (HR) of 1.33. In [138], the authors analyzed data of 6000 COVID-19 patients and found that there was a j-sharped curve between obesity and mortality. Another study was conducted in Latin America [139] and reported a higher risk of infection for people with a body mass index (BMI) > 30 kg/m^2^. This rate increased in lower-income people who already have a higher risk of complications due to healthcare shortages.

#### 2.4.5. Smoking

Smoking destroys the lungs and weakens the immune system [140], so fighting off respiratory diseases such as COVID-19 is hard [141] in smoking people. According to a WHO scientific report [142], around 9.7% of COVID-19 patients are active smokers or have a smoking history. By giving up smoking, you are giving your lungs the chance to become clean and be repaired, improving the ability of a faster recovery. In [143], the authors surveyed the association between smoking, history of smoking, and COVID-19 severity. The study analyzed 16 articles that serve that relation. They concluded that there is a higher association between people who have a history of smoking and COVID-19 infection (odds ratio (OR) = 1:51; 94% CI: 1.11–2.04; *p* < 0.008), between active smoking and COVID-19 infection (OR= 2:18; 94% CI: 1.27–3.45; *p* < 0.001). In another study [112], the authors compared different smoking histories (active smokers, not smokers, and smoker quitter). They reported that 19.07% of COVID-19 patients are smokers.

#### 2.4.6. Medical Comorbidities

Many reports found a high association between COVID-19 and other severe diseases, such as diabetes, hypertension, acute kidney injury, etc. In [133], Wang et al. conducted a meta-analysis study including 1570 patients with COVID-19 infection. The study indicated that patients with serve illness were more likely to have respiratory diseases (OR = 3.42 (1.89 to 6.11)), hypertension (OR = 2.66 (1.46 to 3.82)), and cardiovascular disease (OR = 3.44 (1.44 to 3.82)). Another study [144] analyzed the risk factors of death among COVID-19 patients. The study reported negative markers between COVID-19 infections and other chronic diseases, such as diabetes (33.31%), hypertension (35.16%), chronic kidney disease (17.87%), and diseases of the circulatory system (22.53%). They also compared the death rate among COVID-19 patients and other chronic disease patients. They reported a mortality rate of 22 times higher for kidney disease patients, 10 times higher for patients with hypertension, and 14 higher times for patients with diabetes. Table 6 shows the correlation between medical comorbidities and risk of COVID-19 infection according to the WHO reports [145].

Other researchers focused on analyzing organ complications due to COVID-19 infections. For example, in [146], the authors surveyed organ complications study and showed that about 3.75% of COVID-19 patients reported abnormalities in liver enzymes, 10% developed acute kidney injury, and 23% were afflicted with heart problems. Researchers in [147] developed a DL model to analyze the relationship between mortality and other medical comorbidities. They concluded that medical comorbidities are highly associated with mortality, with percentages of 2.56%, 10.3%, 41.0%, and 6% for heart rate problems, respiratory disease, hypertension, and diabetes; the same trend was found in [148,149,150,151,152]. More details about the correlation between comorbidities and severe diseases are available in [153,154].

#### 2.4.7. Environmental Factors

Several studies addressed the relationship between environmental factors and COVID-19 spread of infection. For example, Aabed et al. [155] investigated the impact of weather, population density, and intra-provincial traffic. They found a positive correlation between infection rate and population density and a negative correlation with social isolation and temperature. The same results were found in [156]. Others focused on investigating the effect of building operation factors, and they found that most infections occurred in an indoor environment [157]. Another critical factor that influences the spread and course of the disease is the possibility of having rapid access to diagnosis. These difficulties may be found in developing countries and in urban areas with high population densities, where the use of public transport and the prolonged frequentation of indoor environments lead to the spread of contagion. These scenarios of inadequate health coverage have been mapped, comparing the quality of access to care with the general conditions of development of the territory [158].

### 2.5. Using DL in Developing Vaccines

Since the outbreak of COVID-19, clinicians and virologists worldwide urged to fight this pandemic ubiquitously, searching for drugs or vaccines with precise and accurate operations. It got even worse with the significant increase in infections [159]. Unfortunately, drug discovery using traditional technologies is a complex process known to take many years. AI techniques can reinforce and improve traditional technologies by accelerating drug discovery, screening, and validation. AI also can speed up the pace by extracting useful data for drug repurposing [160]. The following subsection details the role of AI in drug repurposing, discovery, and vaccine discovery.

#### 2.5.1. Drug Repurposing

**Figure 3:**
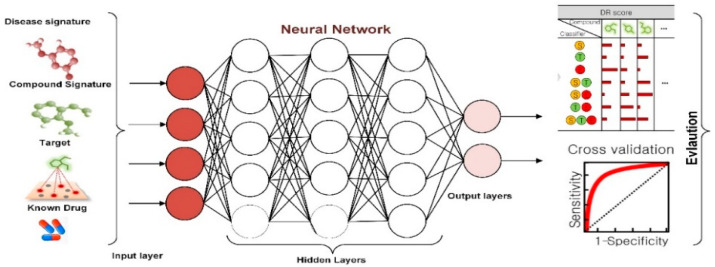
Drug repurposing based on AI techniques.

Drug repurposing is an effective solution in mitigating pandemics, which are based on previously approved drugs. This contributed to rapidly increasing the response against that pandemic and accelerated the clinical trials [161]. Therefore, it is considered the best solution to yield an effective and faster drug against COVID-19 [162]. Several studies [163,164,165] utilized ML and DL techniques, including LSTM, CNN, etc., to search for acting antivirals among the previously known drugs. Four main approaches, namely, docking simulation, ligand prediction, gene expression, and biomedical knowledge graphs (BKGs), have been developed to achieve this goal. The following subsections discuss these four approaches in detail. Figure 3 shows the general method of using AI in drug repurposing.

##### Biomedical Knowledge Graph

BKG is a basic technique that is used to aggregate data from heterogenous resources [166,167,168]. It also is used to capture the relation between entities such as viral proteins and drugs, a pair of genes, etc. For example, Richard et al. [169] utilized BKG to identify Baricitinib. Baricitinib is a drug used in arthritis therapy and is considered a promising treatment for COVID-19. This is because Baricitinib inhibits the protein kinase enzyme, which makes it difficult for the virus to infect the hosted cells. Recent studies showed two main techniques for graph construction. First, in [170], the authors utilized a pipeline of three-part neural network and tree search approach to understand the interaction between all molecules. Second, in [171], the authors utilized BKG to describe the relations between the gene–disease pairs. Others, in [172], utilized ML and statistical analysis techniques to integrate and mine many BKG, showing a relation between the viral protein, human protein, and previously known drugs. These graphs have been used to predict the effective drug candidates against COVID-19.

In [173], the authors extracted 2045 human proteins, which are known drug targets extracted from DrugBank. Then, a multitask ML model was then used to determine the relationship between the known drug targets (KDTs) and the COVID-19 circuits that conform to the diseases. The results showed that 380 KDTs have a direct relation with circuits of the COVID-19. In [174], the authors used a deep graph neural network to extract the candidate drug representation according to biological interactions. They demonstrated that the interactions between DNN and extensive interaction could facilitate the identification of candidate drugs. In [175], the authors utilized an integrative DL model to discover candidate drugs named CoV-KGE. First, the authors built a list that includes 15 million edges from 39 types of relationships, which were extracted from 24 million PubMed publications. They concluded that CoV-KGE had a high performance in identifying repurposable drugs, with an AUROC = 0.85.

##### Protein–Ligand Prediction

Ligands are molecules that bind with protein signals. In [176], the authors used multitask neural networks to predict affinities based on a database of 4600 various drugs—the developed model results in identifying 10 promising drugs with their affinity scores. In similar research [177], authors used a CNN model to identify the inhibitors of the 3C-like protease (the main protease in coronavirus)-based binding DB (BDB) [178] to find an effective treatment for this protein. In [179], the authors also developed a template model of the 3-C like protease, and then applied a mathematical DL model to identify its inhibitors. This model relied on two different datasets (84 SARS inhibitors from chEMBL DB and 15,843 protein affinities from bind DB) [178]. The study resulted in identifying a list of promising COVID-19 drugs from the DrugBank DB.

##### Molecular Docking (Docking Simulation)

Docking is another approach that has been used for drug repurposing, in which each ligand interacts with all proteins in different conformations and orientations. This results in the generation of several poses (known as binding modes). These poses are then utilized to predict the ligand’s affinity [178]. Since these docking simulation techniques are computationally expensive, some studies tried to narrow the pool of candidates that need to be docked using ML and DL techniques. For example, in [180], the authors trained a neural network on 3 million candidates (3-C like protease inhibitors) extracted from 1 billion compounds in ZINIC DB using a deep docking platform. Then, the authors docked the result and presented only the first 1000 results. In another research, Btra et al. [181] trained a random forest model on the SMILES dataset (https://2019-ncovgroup.github.io/data/, access date: 10 February 2021) and applied the docking simulation, which resulted in identifying 187 molecules in the coronavirus S-protein. In [182], the authors proposed an ML framework that is used to predict viral protein activity. This was done by developing an ensemble model that ranks the drugs according to their ability to inhibit the SARS-COV-2 virus proteases. The developed model helped in identifying 19 drugs (7 antiviral, 3 antibodies, 6 anticancer, 1 antifungal, and 2 antimalarial). Then they use molecular docking to evaluate the binding ability. They concluded that antiviral and antimalarial drugs have more binding energy with 3CL pro protease than anticancer and antibiotic drugs.

##### Gene Expression Signature

Studies discovered therapies that have a similar impact to other previously known treatments depending on gene expression signatures. Avaachuv et al. [165] utilized this approach to find a gene expression signature similar to COBP2, limiting COVID-19 replication. The study resulted in 20 promising drugs, many of which have been previously used as antivirals [183]. Since all these drugs already got clinical approval, they may facilitate the discovery of an effective treatment.

#### 2.5.2. Drug Discovery

Another role of AI in COVID-19 treatment is to discover new chemical compounds, using ML and DL models to identify baricitinib to tackle COVID-19 [161]. For example, Zahavorkov et al. [180] tried to find inhibitors for the 3-C like protease. They used three main inputs, include co-crystal ligands, a crystal protein structure, and the protein homology model. In total, 28 different models were trained for each input (i.e., generative adversarial networks and generative autoencoders [180]). The authors then used reinforcement learning with reward functions to evaluate the drugs according to different factors (i.e., novelty, diversity, etc.), to confirm choosing the most suitable molecules and thus guaranteeing to find a novel drug. Reinforcement learning has also been used in another study for drug discovery [184], where the authors used a list of 183 molecules known as inhibitors for SARS, breaking these proteins into 315 fragments. Deep Q learning was used then to combine fragments based on fragment drug design (ADQN-FBDD). This design scored the discovered molecules based on three points (drug-likeness, the existence of known pharmacophores, and the presence of pre-pet-determined fragments). The 4900 molecular were filtered using a heuristic filter to choose the promising compounds [180]. Similarly, in [180], the authors used 1.6 million molecules extracted from the chEMBL dataset [185] and generated 33 candidate inhibitors. Other researchers took a different path to discover a new drug for COVID-19, which depended on the immune response. In the human body, B-cells produce antibodies (known as antigens) that attack the virus. As such, researchers tried to discover new drugs by searching for antigen-neutralizing antibodies. For example, in [180], the authors created a dataset of 1933 antigen sequences from similar diseases (SARS, HIV, and EBOLA); then, they trained the XGBoost model (classification model) to predict the antibody that will affect the antigen. Other researchers [186] tried to predict effective anti-bodies from the future generation of COVID-19. They mutated the SARS antibody sequence and generated 2900 antibody sequences. Then, these mutations were filtered to choose the stable variants and propose the effective antibodies.

#### 2.5.3. Vaccine Discovery

From the medical side, the human body attack viruses in two ways: (1) via B-cells that produce antibodies (as described above); and (2) via T-cells. T-cells include small cells called memory cells, which could recognize the antigen quickly, and then activate more T-cells to attack the virus directly [187]. A part of the immune system is the complex proteins (MHC I and MCH II), which shows the binding areas with the antigens (known as epitopes); these proteins are encoded by Human Leukocyte Antigen (HLA) genes, and vary from human to human [187,188]. On these bases, the vaccine should identify the suitable epitopes and ensure that these epitopes could be presented by MCH I and II genes generated from different HLA [189]. Altman et al. [190] identify 405 T-cell epitopes that could be presented by MHC I and II proteins. They utilized a previously trained neural network to predict the T-cell epitopes that could present with MHC genes. To assure choosing the potential epitopes, the authors examine 68 genetic variants of the SARS-COV virus to analyze the mutation of the virus, to identify the areas of the virus that are more or less likely to mutate [191,192]. They concluded that S-protein is the most suitable part for the vaccine, as it does not include too many such mutations. In another research [193], the authors used an XGBoost model to predict the best protein that could serve as an effective vaccine. They reported that the six proteins (i.e., nsp3, nsp4, nsp5, nsp6, nsp7, and nsp8) are also promising for vaccine development, in addition to the S protein. As far as we know, three different vaccines (clinically approved vaccines) reported that they used ML in their development process [189]. However, it is discouraging that the developed companies published minimal information about their methodologies pipeline and how they integrate ML into the vaccine development pipeline.

### 2.6. Applications of AI to Support COVID-19 Patients

ML and DL have been extensively used in various and critical health care applications, such as predicting brain age [194], diagnosis of liver diseases [195], and many other diseases [196,197]. In the current pandemic, governments and healthcare organizations are in critical need of support and decision-aid tools, which may help get timely and efficient support to avoid virus spread. AI tries to provide professional solutions that mimicked human intelligence and results in various significant applications that could be used in screening, diagnosing, and tracking the disease. This section concentrates on AI applications that gained much interest and raised the world’s hope to fight against COVID-19. AI is used to tracking patients through smart devices, such as mobile phones, cameras, and other wearable sensors [198,199]. These devices could be used for diagnosing, screening, and continuous monitoring [200]. Based on data aggregated from these devices, AI could provide useful information for the decision-making process, such as prioritizing the need for respiratory support as well as intensive care unit (ICU) admission [58,201].

Several AI applications have been developed to lighten the burden on medical experts as well as healthcare workers. This is done by automating procedures in a way that minimizes their direct contact with patients as follows. (1) AI is used to analyze patient’s data (i.e., symptoms, clinical reports, etc.), and to classify them into different categories, such as mild, moderate, and serve. Accordingly, different therapy plans can be adopted for patients efficiently. (2) AI telemedicine applications could help in reducing the frequent visits to hospitals by providing continuous monitoring for patients with mild symptoms [202]. (3) Another application that supports both patients and health care staff is the AI-based medical chatbots (i.e., Clara chat boot 44). Chatbot is an AI service that is incorporated with ML and DL models (i.e., feature extraction, NLP, etc.) to assist patients with instant answers, providing continuous guidance on how to deal with potential problems. From the health care organizations’ side, chatbots could assist in triaging patients to flow smoothly, automate primary care, and allow medical experts to focus on critical and dire cases [203,204,205]. (4) AI is used as the core of service robotics that could assist in several tasks, such as cleaning, disinfecting, delivering food, and treatment [206,207,208]. Moreover, depending on AI to understand population awareness towards COVID-19 through social media could help in specifying the correct strategy for mitigating this pandemic. ML and DL were utilized to make a sentiment analysis towards the followed strategies, recognize trends, and determine the origin of such misinformation and rumors [35,209,210]. AI could also help analyze the updated information, such as the recovery rate and therapeutic results, which may help medical experts resolve panic and fear towards this pandemic [131]. More applications that utilized AI techniques to support or monitor COVID-19 patients are expressed in Table 7.

## 3. COVID-19 Datasets

The lack of accurate and sufficient data is one of the key problems in COVID-19 research, as the number of carried-out tests is small, and thus numerous death and infected cases are left unreported. No country worldwide has succeeded in offering reliable and accurate datasets to the virus’s existence among their population. However, the research on this context cannot stop. Therefore, information fusion has a significant role in combining information from multiple sources. Information fusion is used to integrate data from various resources to provide valuable information for the characterization, identification, and detection of a specific entity [233]. Given the fact that in ML and DL models the existence of a large size dataset plays a key role in developing models with high prediction accuracy, the datasets of COVID-19 were categorized into three main groups: (1) textual data; (2) medical images; and (3) speech. Most COVID-19 image datasets were taken from screening tools that belong to three main classes, namely, X-ray, ultrasound, and CT chest scans. As the kits used in the PCR test are timely, limited, and costly, medical images are considered an adequate alternative that lower the burden on PCR tests.

### 3.1. Medical Images Datasets

Medical images, such as X-ray and CT chest scans, were used to develop an automated model for disease diagnosis. Datasets often need preprocessing steps, such as segmentation and augmentation [25]. Image segmentation leads to portions of the image (region of interest). Image augmentations include transformation and filtering to increase the size of the dataset [42]. Consequently, ML and DL provide accurate models and avoid overfitting. The following subsections discuss the available medical image datasets for COVID-19.

#### 3.1.1. CT Chest-Scan Dataset

**Figure 4:**
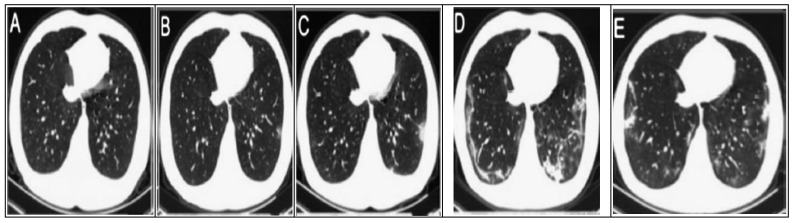
(**A**–**E**) subfigures show progression of a CT scan of a COVID-19 patient across days (2, 4, 5, 6, and 8, respectively).

Owing to the rapid progression of the COVID-19 disease, a subsequent CT scan every 2–4 days is required to evaluate the progression and therapeutic effect. Figure 2 shows the changes in CT chest images of the COVID-19 patient, which took place gradually [28,35,234]. Initially, there is a slight change in the chest CT images; but, as infection rises day by day, bilateral differences are seen to take place. Chest CT images clearly show the growth of pneumonia with linear opacity in the subpleural area [235]. Figure 4 shows the progression in the patient’s status.

A pioneering effort in collecting public CT scans datasets was in [236]. The dataset consists of 125 chest CT scans. It includes images of several classes (COVID-19, SRAS, MERS, and ARDS). The dataset was collected from several websites and publications, which may affect the image quality and even the performance of the ML model [237]. Another published CT chest dataset is in [25]. It includes 275 images of positive COVID-19 CT scans extracted from 760 COVID-19 preprints. The dataset is used in various studies and updates continuously in the online repository. To overcome the shortage in COVID-19 datasets, several studies use augmentation and segmentation techniques to increase the size of the dataset. The segmentation is considered a preprocessing step used to crop the region of interest (infected region). For example, in [34], the authors use a 3D CNN model to segment the infected regions from the CT chest scan dataset [236]. The system made auto-contouring to estimate the shape and percentage of the infected region, resulting in an accuracy of 90% in recognition. Other segmented datasets are listed in [238], consisting of 20 labeled COVID-19 datasets categorized into left and right infected lungs. Another COVID-19 online dataset is available at http://medicalsegmentation.com/covid19/, access date: 10 February 2021, the segmented images obtained from a society of medical and interventional radiology (SIRM) (https://www.sirm.org/en/category/articles/covid-19-database/, access date: 7 February 2021; https://coronacases.org, access date: 10 February 2021) and categorized into three classes (consolidation, pleural effusion, and ground glass). Another effort for collecting a COVID-19 dataset is in https://coronacases.org/, access date: 10 February 2021. The UK imaging and British society of thoracic imaging developed an online portal for COVID-19-positive CT-scan images (https://www.bsti.org.uk/training-and-education/covid-19-bsti-imaging-database/), access date: 10 February 2021. Each case is stored with its characteristics, such as gender, age, and PCR result test. The same procedure was done to collect the dataset in https://www.sirm.org/en/category/articles/covid-19-database/, access date: 10 February 2021/Several studies utilized these datasets in their research [18,239]. To make a binary classification for COVID-19 identification and diagnosis, several studies use non-COVID-19 CT chest-scan images as a negative training example, such as the following: (1) the MedPix (https://medpix.nlm.nih.gov/home, access date: 10 February 2021) medical images dataset that includes 5900 images for 1200 patients; (2) the LUNA (https://luna16.grand-challenge.org/) dataset for lung cancer patients that includes 888 CT chest scans for 888 subjects; and (3) the Radiopaedia online repository (https://radiopaedia.org/articles/covid-19-4?lang=us, access date: 10 February 2021) that includes 366,558 CT scan images.

#### 3.1.2. X-ray Images Dataset

**Figure 5:**
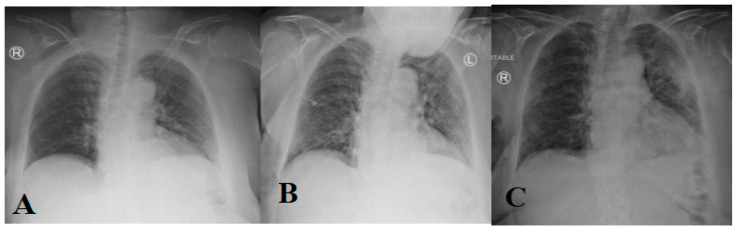

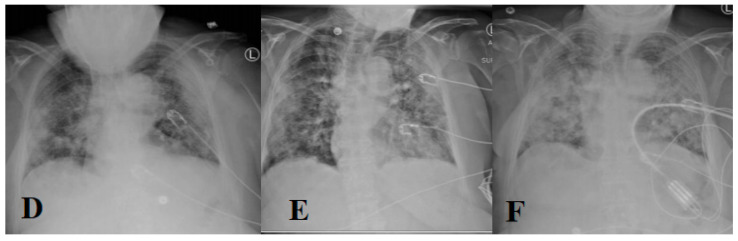
(**A**–**F**) subfigures show progression of an X-ray image for a COVID-19 patient across days (1, 3, 6, 7, 8, and 10, respectively).

A chest radiograph (X-ray) is the common way to diagnose patients with respiratory diseases. A chest X-ray image can be viewed as normal at the early stages, but it gradually changed in a way that may correlate with other respiratory diseases such as pneumonia or acute respiratory distress syndrome (ARDS). Two common changes that arise in the COVID-19-infected lung include (1) accumulation of tissue or fluid in a way that prevents gas exchange; and (2) the appearance of nodular shadowing. Figure 5 shows the progression of X-ray images for a 45-year-old patient.

An earlier effort to develop an X-ray dataset for COVID-19 patients was in [240]. It includes 13,800 images for 13,000 patients collected from several online repositories. Wang et al. [240] collected this dataset to develop a CONVNET model for COVID-19 diagnosis, resulting in a classification model with an accuracy of 93.11%. Another dataset collected from online repositories by Cohen et al. [236] continuously updated through the following link (https://github.com/ieee8023/covid-chestxray-dataset, access date 12 February 2021). Several researchers utilized the Cohen X-ray images dataset in their studies. For example, Hemdan et al. [58] utilized Cohen et al.’s dataset [236] to develop a CNN model for COVID-19 diagnosis. They developed five different DL models based on transfer learning to overcome the shortage of the dataset. Other researchers merged Cohen’s [236] dataset with other datasets to increase the size of the resulting dataset to enhance the performance and avoid overfitting. For example, in [241], the authors merged the Kaggle dataset (https://www.kaggle.com/andrewmvd/convid19-X-rays, access date: 14 February 2021), for pneumonia with the Cohen dataset [236] to train a CNN model using pre-trained models, including VGG19, Inception, Xception, MobileNet2, and ResNet V2. Results show that MobileNet V2 outperformed other models in terms of accuracy, specificity, and sensitivity. The authors extended their study in [215] by merging Cohen’s dataset [236] with SIRM and RSNA [241] data, where a total of 455 images were obtained for all classes. This research demonstrated that building the CNN model from scratch based on a sufficient dataset outperformed transfer learning. In another research [24], Cohen’s dataset [236] was merged with the Kaggle dataset (https://www.kaggle.com/paultimothymooney/chest-xray-pneumonia, access date: 14 February 2021) and resulted in a 100 CT image dataset that was divided into two balanced classes (50 normal and 50 positive). Apostolopoulos et al. [53] used the same dataset and merged it with the Kaggle dataset (https://www.Kagglee.com/andrewmvd/convid19-X-rays, access date: 10 February 2021). This resulted in 127 images from pneumonia and COVID-19 cases. In [213], the authors utilized the augmentation techniques on Cohen’s dataset [236] in resolving the COVID-19 data scarcity. The same has been done in [242], where authors applied data augmentation techniques on COVID-19 and non-COVID-19 X-ray images. They obtained around 17,000 X-ray images from 4044 positive images and 5500 negative images. The same was done in [243], where the authors utilized both the Cohen dataset and Kaggle dataset at (https://www.kaggle.com/paultimothymooney/chest-xray-pneumoni, access date: 16 February 2021). The authors used data augmentation techniques and obtained 2500 images (1340 viral pneumonia and 190 COVID-19 images). Data after augmentation is available at (https://www.kaggle.com/tawsifurrahman/covid19-radiography-database, access date: 14 February 2021). In [244], Signoroni et al. collected 4707 X-ray images for COVID-19-positive subjects collected from an Italian hospital. To maintain a robust dataset, the authors collected it from two different modalities, including (direct X-ray (DX) and computed radiology (CR)) for patients with various statuses (i.e., supine, standing, and with or without life support systems).

Notwithstanding the importance of X-ray in the diagnosis of COVID-19, X-ray chest images are unreliable at the early stages of COVID-19 disease [245]. In other words, the reliability of X-ray findings mainly depends on the difference in time between the first symptoms and the imaging procedure. An Italian study, conducted in April 2020 on 72 COVID-19 patients [246], reported that the disease is visible on an X-ray image within the first 4 days after the onset of the initial symptoms, such as a dry cough, fever, etc.

#### 3.1.3. Ultrasound Dataset

**Figure 6:**
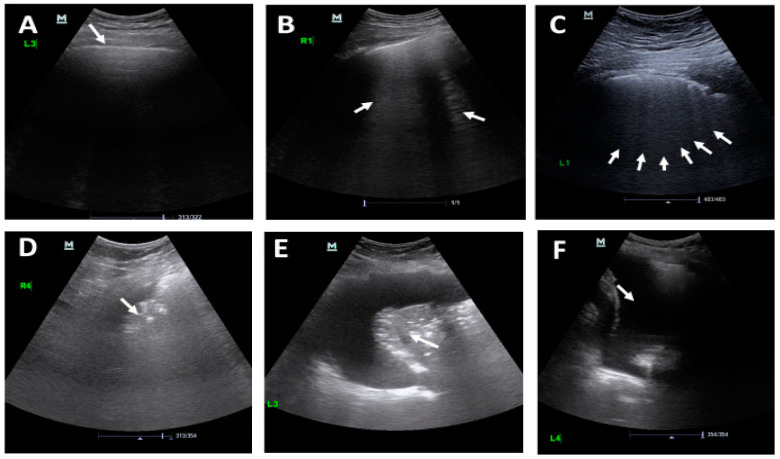
(**A**–**F**) subfigures show the progression of a US image for a COVID-19 patient across days (1, 3, 6, 7, 8, and 10, respectively). The white arrows in each subfigure clarify the change in each day.

Lung ultrasound correctly diagnosed COVID-19 in 96% of people with COVID-19. However, few US datasets are available. For example, in [70], the authors aggregated a dataset of 64 videos that were divided into 39 videos of COVID-19 and 15 videos of pneumonia, and 12 videos for healthy patients. Another dataset available at (https://tinyurl.com/yckfqrcg, access date: 17 February 2021; https://pocovidscreen.org/, access date: 16 February 2021) includes 1101 ultrasound images and is categorized as 650 images for COVID-19, 276 for bacterial pneumonia, and 171 for healthy cases. These images were extracted from different videos published in research works. Figure 6 shows the progression of a US image for a COVID-19 patient.

### 3.2. Sound Dataset

The main challenge in developing such modes is the shortage of available datasets. The earliest and noteworthy have been developed in [80,247], known as the Coswara dataset (https://coswara.iisc.ac.in/, last access date (16 March 2021). Coswara is a public dataset collected via public media interviews. Since writing this paper, Coswara included 102 records for breathing and deep cough sounds aggregated from COVID-19-positive patients. The collected data include shallow and deep cough sounds and slow and fast breathing sounds. Gender, age, health status (i.e., infected, cured, or exposed), and geographical information are also stored for each patient. Another cough dataset [248] was collected in South Africa, known as SACRO (https://datahub.io/core/covid-19, last access 22 March 2021) (SARS COVID-19 South Africa). SACRO is a small dataset collected from 21 cases (8 COVID-19 cases and 12 healthy cases) through a smartphone. Cough sounds were collected, and then sampled at a 44.1 sampling rate. Age, gender, county, COVID-19 lab test result (positive or negative), and symptoms were also recorded in addition to the cough sound. Due to the imbalance in the SARCO dataset, the authors used the synthetic minority oversampling technique (SMOTE) [249] to make the data balanced before utilizing it in detection and classification processes. In [250], the authors collected 260 sound samples from 52 COVID-19-positive cases via the WeChat app. They recorded five sentences one after the other via the mobile app for each patient. These sentences were analyzed to specify the degree of anxiety, fatigue, sleep quality, breath rate, etc. In another dataset [76], the authors collected 7000 sound samples that included 200 confirmed COVID-19 subjects.

### 3.3. Text Dataset

**Figure 7:**
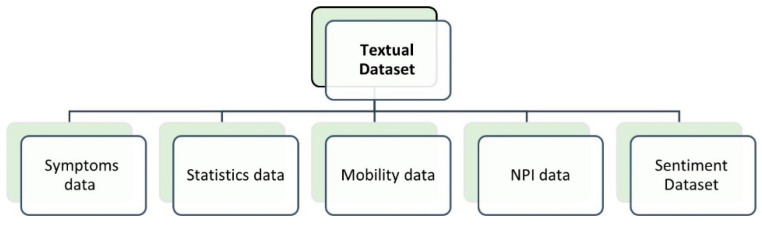
Types of textual datasets.

Since the COVID-19 pandemic, various textual datasets have been developed with different targets. It could be categorized as following: (1) reporting and visualizing COVID-19 cases in time-series formats; (2) measuring the community transmission; (3) correlating the effect of mobility on virus transmissions; (4) evaluating the impact of (non-pharmaceutical interventions) NPI on COVID-19 cases; and (5) analyzing COVID-19 scholarly publications for semantics. The categorization of the textual dataset is shown in Figure 7.

The earliest dataset that was developed to aggregate COVID-19 statistics summarization (number of infected recovered and death grouped by county) can be found in [251]. It was developed by Johns Hopkins University, where a real-time dashboard (https://www.arcgis.com/apps/opsdashboard/index.html, last access date (16 March 2021). was developed to aggregate data. These data are publicly available at (https://datahub.io/core/covid-19, last access date (16 March 2021). The main objective of this dataset is to provide the health authorities as well as researchers with statistical data that could be used to analyze, track, and predict the spread of the COVID-19 pandemic. The Chinese Center for Disease Prevention and Johns Hopkins University developed another time-series dataset, which includes the number of recovered and infected cases, the time of infection, and the origin county. Other researchers [252,253] provided an epidemiological dataset about COVID-19 cases in China. This dataset includes personal and laboratory information, such as demographic data, disease onset date, admission date, last travel date, etc. It is updated continuously to guide public health in the decision-making process. In [254], the authors provided a textual dataset that includes four time series datasets: (1) the daily infected cases in Wuhan; (2) the daily internationally exported cases; (3) the daily infected cases in China; and (4) the percentage of the infected cases on vacation flights. This study aimed to estimate the transmission of infection, the virus outbreak, and the effect of travel bans on infection transmission. In the same manner, in [255], authors utilized the daily case reports to evaluate the impact of travel restrictions on COVID-19 spread, where in [256], the authors used case reports that were collected from location-based systems (i.e., WeChat). In [257], the authors analyzed the effect of mobility and travel restrictions on spreading COVID-19 in China. The authors developed a dataset that includes real-time and historical data aggregated in Wuhan, China, in addition to the list of cases inside and outside Hubei, available at https://github.com/Emergent-Epidemics/covid19_npi_china, last access date (16 March 2021). This study found a high correlation between the spatial distribution of COVID-19 and mobility. Another study utilized an epidemiological dataset extracted from government websites and official sources [258] to evaluate the effect of travel restriction on limiting the spread of infection.

Another research interest is concerned with studying the effect of NPI restrictions. NPI is a wide range of rules and restrictions applied by the government to fight against the COVID-19 pandemic (i.e., social distancing, travel limits and bans, contact reduction, etc.). Such datasets are essential to show the effect of applying NPI on infection transmission. At Oxford University, a team of academic researchers started the Oxford COVID-19 government response tracker (OxCGRT) project, which includes data from various countries in the Stringency Index [259]. The Stringency Index consists of 17 indicators, such as local and international travel bans, contact tracing, cancelling all public events, etc. These indicators are utilized to compare the government response, the public awareness, and the effect on the transmission rate. The aggregated data are available at a GitHub repository (https://github.com/OxCGRT/covid-policy-tracker, last access date: 16 March 2021). Another dataset aggregated by a group of volunteers can be found at https://www.kaggle.com/davidoj/covid19-national-responses-dataset, last access date (16 March 2021). The main objective of this is to analyze the effect of NPI regulations in 117 countries, regardless of economic factors. Unfortunately, the authors reported that the data might be biased to some countries, as some countries are not concerned with the document, and their actual implementation may differ from the basic reports.

It is essential to understand the emotional, public response, and worries towards the COVID-19 pandemic in this global crisis. The earliest effort in this regard was in [260], wherein authors requested various participants to report their emotions and developed a dataset of tweets (short and long tweets) aggregated from 2500 participants. The authors also asked the participants to rank their feelings using nine points, to gauge the anxiety, anger, relaxation, happiness, and sadness they felt. In another large-scale tweet dataset, the authors used Twitter API stream to aggregate tweets that include specific keywords (i.e., COVID-19, pandemic, SARSCOV, etc.) [261]. They aggregated 434 million tweets. Twitter streaming API was also used to collect a dataset of Arabic tweets [262]. These data aimed at analyzing the Arabian countries’ behavior towards the pandemic, and authors collected 2,433,660 tweets in addition to the geolocation of the tweet.

### 3.4. Genome Sequence Dataset

Genome sequencing is critical to specify the order of chemicals inside DNA molecules and identify virus gene expression [1]. Virology scientists utilized these sequence data in the processes of vaccine development or mutation recognition. During the early breakout of the pandemic, there were a very limited number of genome datasets in Wuhan, China. The lack of genome transfer data made the virus analysis more challenging and raised doubts on virus recombination and phylogenetic network results. With the rapid increase of COVID-19 in different countries, several studies reported that the virus had accumulated several alterations of genome sequences, which have been seen in the spread of viral strains [163]. Until now, more than 66,000 viral genome sequences have been shared through the global initiative on sharing avian influenza data (GISAID) (https://www.gisaid.org/, last access date (16 March 2021). [263]. The availability of the mutated genome sequence raises the chance to discover new drugs and vaccines. Several datasets have been developed for this purpose. In this study [1], the authors developed a stream of virus sequence datasets that included two types of data (raw data and processing data). The raw data had 1557 instances of the SARS-COV-2 virus genome that was collected from NCBI and 11,540 collected from another virus-host, in addition to three other virus sequences (bat-SL-COVZC45, bat-SL-COVZC22, and RAT13). These viruses had a large similarity with the SARS-COV virus. The processing part consists of various types of data stream representations (DSRs), including direct mapping and k-mers mapping with Chaos Game Representation (GCR). Another centralized repository of virus sequence included both the original coronavirus sequence available at (https://registry.opendata.aws/ncbi-covid-19/, last access date (16 March 2021).

Other projects were developed to aggregate virus mutations. For example [264], the VIPR project was a pathogen platform that provided the ability to search and download information about virus mutation. However, it lacked the connecting information between virus mutation, country, and time of occurrence, which is essential to analyze the transmission path. The main objective of such projects was to give users the chance to analyze virus mutations from different perspectives. Table 8 includes a summarization of all the COVID-19 datasets from different angles.

## 4. Discussion

**Figure A1:**
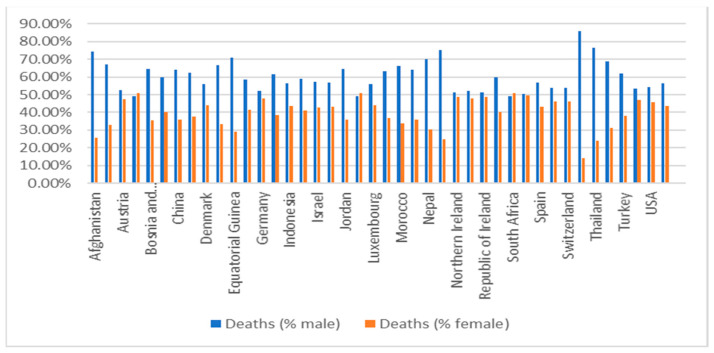
Distribution of infected people in terms of gender (male, female) among various countries.

The dramatic spread of the COVID-19 and the consequent increase in the number of medical examinations throws a heavy burden on healthcare organizations. This is due to the shortage of medical expertise and test kits. That is why AI is considered a forefront tool to face the COVID-19 outbreak. Recently, several papers focused on surveys of the COVID-19 state of the art from different perspectives. For example, in [267], the authors surveyed the usefulness of the prediction models for COVID-19 diagnosis. In [268] and in [242], the authors briefly summarized the deep learning applications that were developed to combat COVID-19. Same in [269], where the authors summarized the state of the art in medical image processing and its significant role in the COVID-19 domain. Another survey focused on the role of transfer learning. The main differences between our study and other surveys in COVID-19 are the following: (1) investigated the role of AI in the COVID-19 pandemic; (2) covering all applications from diagnosis using various medical datasets; (3) understand the current spread of the pandemic state and predict future spread; (4) specifying the correlation between COVID-19 infection and other healthcare factors; and (5) surveying the role of AI in developing drugs and vaccines. Table A1 show the distribution of gender, ages, and death rate among various countries. Figure A1 show this distribution graphically We tried to analyze how the progress of deep learning contributes to combat coronavirus by developing effective solutions.

First, we compare studies that are concerned with using AI in COVID-19 diagnosis through medical images. Based on this comparison, we observed that (i) a large number of studies have utilized CT scans and X-rays in their works [243,270,271], where few studies utilized lung US [55,66,272]; (ii) although X-ray chest scans are considered less sensitive than PCR tests in detection of COVID-19 at the early stages, it is recommended for monitoring and evaluating the progression of a patient’s status, especially with critical cases [215]; (iii) segmentation techniques that used to detect the infected region are primarily used in CT scans [273]; (iv) augmentation techniques that used to increase the size of the dataset are commonly used with X-ray datasets [274]; (v) the majority of COVID-19 studies utilized CNN in their classification process [52,275], where some of them integrate CNN and transfer learning to overcome the shortage of the available dataset and increase the accuracy of the model [32,201,276]; (vi) a small number of studies augmented CNN with random forest and support vector machines to make feature extraction and classification [277,278]; (vii) higher accuracy reported from studies that augmented CNN, transfer learning, and SVM, where using CNN and DL are reported to overfit in some studies due to the shortage of available datasets [37,162]; (viii) accuracy of diagnosis using X-rays in diagnosis is approximately equal to the accuracy when using CT chest scans; (ix) the sensitivity of X-ray in diagnosis is highly correlated with the difference between the time of the initial symptoms and the procedural images;—it was not more than 55% after 2 days from the initial symptoms and increased to 79% after 11 days from the symptom onset [147]; (x) VGG, MobileNet, and ResNet are the most commonly pre-trained models employed for the classification tasks [21,52]; (xi) explainability of CNN model have been rarely used in clarifying the results of CNN [57]; and (xii) most of the studies reported accuracies of more than 90% for the binary classification tasks (i.e., COVID-19, non-COVID-19) [218,279], and reported accuracies higher than 80% for three classification tasks (i.e., normal, viral pneumonia, and COVID-19) [216,280]. Table 2, Table 3 and Table 4 present summarizations of the many studies that used medical images in COVID-19 diagnosis.

Second, we concentrated on using AI techniques in COVID-19 diagnosis based on respiratory sounds. Accordingly, we make the following observations: (i) a cough sound has unique characteristics, and therefore could be used to differentiate respiratory diseases in the early stages of the diseases. AI models could effectively learn these features and discriminate between COVID-19 and non-COVID-19 cough sound; (ii) quantity and quality of the respiratory sound datasets are the main challenges that face AI in providing robust prediction; (iii) the majority of COVID-19 sound datasets have been aggregated by volunteering the general population through mobile apps and websites. Therefore, prescreening tools are essential to build effective models.

Third, we focused on textual datasets and their role in fighting against COVID-19. We observed that (i) a textual dataset is used for several purposes, including reporting several infections in time series format, correlating the NPI and lockdown effect with virus spread, estimating the reproduction and mortality rate, and analyzing social media data for semantics) [136,189,281,282]; (ii) extracting human emotions towards the pandemic and the NPI from articles and social media data are not deeply investigated; (iii) most research that worked on social media data did not consider the timeliness of the study, as such data got outdated quickly [242]; (iv) contact tracking application is very limited due to the difference in privacy and security regulations across different countries [246,254]; and (v) several papers were written in the Chinese language, especially papers published during the first stage of the COVID-19 pandemic. Thus, it may not be useful for many researchers.

Finally, we compared all COVID-19 available datasets, make several observations. First, regarding the medical images dataset, (i) several studies did not publicly include their data and code. Therefore, we cannot reproduce the results of the research conducted with these data [264,265]; (ii) other studies aggregate data from several resources, but they did not host it in a new repository; and (iii) augmenting data may help in solving the data scarcity issue, increase the performance of the model and avoid overfitting—however, the accuracy of using augmented data needs to be evaluated. Second, we observe that real news is much longer than fake news regarding textual datasets in terms of several words per post or article. Table 7 summarizes all the COVID-19 datasets.

## 5. Limitations and Future Directions

This section highlights the most critical challenges in the literature and the possible research directions for future work.

- Symptoms of COVID-19, pneumonia, and other respiratory diseases are very similar, therefore developing a suitable DL model that could detect COVID-19 with optimum accuracy remains a challenge [74].
- The scarcity of a high-quality dataset for COVID-19 is a major challenge. This returns to different reasons, including (1) closed source and non-published datasets; (2) the distributed nature of COVID-19 datasets; and (3) privacy issues that limit data sharing [32]. Therefore, the collaboration between all medical organizations across the globe is essential to expand the existing dataset and accelerate AI research for COVID-19.
- The variability in the testing process across different countries and hospitals is a critical concern that may lead to non-uniformity in the labeling process.
- COVID-19 virus is rapidly mutated over different geographic areas. Therefore, data collected from one region may not be suitable to draw interferences on another region [226].
- Medical staff are considered the first line of defense against this pandemic. Therefore, work on more contact-less screening and diagnosis tools is an urgent need to protect them from infections.
- Most state-of-the-art DL models were trained in 2D images. However, most MRI and CT scan images are 3D, and hence adding an additional dimension is essential to optimize the impact of these images [40,44].
- The non-standardized process when aggregating medical image datasets result in increasing data variety; thus, this raises the need to ensure the robustness of DL-generated models.
- Most of the available COVID-19 datasets are limited in size. Therefore, transfer learning is a future research direction that could help detect abnormalities in small datasets and yield robust predictions and remarkable results [241].
- Based on the literature, it is noticed that there is a correlation between COVID-19 infection and other medical comorbidities. Therefore, to provide a precise and accurate prediction model, a patient’s history of other ailments (diabetes, liver, kidney, heart disease, etc.) must be taken into consideration in both the COVID-19 prediction and detection process [144,145,146].
- High computational resources are required to build complex DL models, processing, and interpreting big data, compared to working with IoT devices. Therefore, edge computing and fog computing could be effective in handling this challenge [199].
- Various preprocessing steps are required to enhance the interpreting data extracted from various sensors (i.e., data cleaning, outlier detection, quality improvement, etc.) [51,260,283,284].
- Current NLP applications have limited the benefit from such a diagnosis system. Therefore, working in algorithms that measure semantic textual similarity (STS) [285] is essential to translate performance to a specific domain environment (i.e., COVID-19).
- Data fusion is a challenge because it integrates heterogeneous data [232]. However, it improves the performance of the resulting models. There are many fusion techniques in the literature. Therefore, adaptive multi-models are highly needed to handle data from multiple sensors [286].
- More sophisticated techniques are needed to optimize the performance of processing X-ray and sound data.
- The explainability and interpretability of ML/DL techniques is a key challenge. ML model should not be a black box. Medical experts must know which features are chosen to distinguish COVID-19 from non-COVID-19 [232]. Moreover, ML/DL should investigate how to predict infections before the symptoms appear.
- Several ML and DL models have shown promising results in COVID-19 screening, diagnosis, and prediction. However, most of these models are not deployed in a real environment (i.e., emerging services, hospitals, etc.) to show their capabilities in tackling the COVID-19 pandemic. Therefore, lots of challenges need to be addressed to deploy such diagnosis models, including (1) addressing the consistency of the network security to provide more reliable communication and trusted data on the network; (2) adaption of cloud, fog, and edge computing; and (3) security and privacy issues regarding the patient’s data that also need to be handled.

## 6. Conclusions

COVID-19 is an ongoing pandemic that outperforms most communicable diseases in terms of death and infection rate. Therefore, medical experts as well as AI scientists are trying to fight against this pandemic and are searching for alternative techniques that could provide rapid tracking, screening, and development of drugs and vaccines. This paper aims to survey recent studies that investigated AI solutions to combat the COVID-19 pandemic. It includes AI solutions for diagnosis, estimation, treatment, and association. This paper also surveyed open-source datasets (medical images, speech dataset, test dataset, and genome structure dataset) and studied the challenges and limitation issues of the current AI literature. Finally, the paper discussed the future direction in terms of data aggregation, data preprocessing, and ML and DL deployment in real environments. The study concludes that ML and AI have dramatically enhanced disease screening, diagnosis, monitoring, and drug/vaccine discovery for the COVID-19 pandemic and minimize human intervention in a way that minimizes burdens on the healthcare sector.

## Figures and Tables

**Table 1:** List of abbreviations.

Term	Abbreviation
AI	Artificial Intelligence
ARDS	Acute Respiratory Distress Syndrome
AKI	Acute Kidney Injury
AUC	Area Under the Roc Curve
BSTI	British Society of Thoracic Imaging
CAP	Community-Acquired Pneumonia
CFRs	Case-Fatality Rates
CNN	Convolutional Neural Network
COVID-19	Coronavirus Disease 2019
CR	Computed Radiology
CT	Computed Tomography
DL	Deep Learning
DX	Direct X-ray Detection
EBI	European Bioinformatics Institute
GISAID	Global Initiative on Sharing Avian Influenza Data
ICT	Information Communication Technology
KSA	Kingdom of Saudi Arabia
NCBI	National Center for Biotechnology Information
RNA	Ribonucleic Acid
RT–PCR	Reverse Transcriptase Polymerase Chain Reaction
SEIQR	Susceptible–Exposed–Infected–Confirmed–Removed
SEIR	Susceptible–Exposed–Infected–Recovered
SIR	Susceptible–Infected–Recovered
SIRM	Society of Medical and Interventional Radiology
OR	Odds Ratio
WHO	World Health Organization
3CLpro	3C-Like Protease

**Table 2:** Diagnosis ML and DL algorithms based on CT scans for COVID-19 patients.

Ref.	Year	Model	Task	Dataset	Evaluation Metrics		
					ACC	P	SN
[40]	March 2020	3D CNN model	Using CT chest images infiltrative biomarkers	498 CT scans from 151 positive COVID_19 subjects and 497 CT scans from different subjects with various types of pneumonia	70.02	-	-
[22]	June 2020	Desenet201 pre-trained model with CNN	Object detection, binary classification	1260 COVID-19 images and 1232 CT from health patients	96.21	96.20	96.20
[28]	June 2020	CNN Model	Binary classification	413 of COVID-19 images and 439 of health images	93.01	95.18	91.45
[24]	May 2020	3D CNN model	Multiclass classification	219 CT scans from COVID-19 patients, 220 from IAVP and 174 from healthy people	83.90	81.30	86.70
[29]	March 2020	Segmentation models (V-Net, U-Net, FCN) and classification models (ResNet, inception)	Detection	732 COVID chest CT scan (400 from normal cases and 332 from COVID_19 cases	92.22	-	97.21
[31]	May 2019	CNN model	Multiclass classification	10,000 CT images related to four classes, including COVID-19, non-viral pneumonia, influenzas, and non-pneumonia	-	95.75	90.11
[35]	March 2020	ResNet-50 model	Multiclass classification	60,457 CT chest scan images were collected from 100 COVID-19 cases, 102 non-COVID-19 viral pneumonia, and 200 normal lungs.	98.81	98.20	94.52
[36]	June 2020	DenseNet121 model	COVID-19 prognostic tool	4106 CT images (925 COVID-19, 342 pneumonia)	78.33	76.61	80.39
[37]	March 2020	Hybrid classification technique (CNN and ML)	Predicting the recurrences in both SARS and COVID-19 cases	51 SARS and COVID-19 CT chest scans from the Kaggle benchmark dataset.	96.20	96.12	96.77
[41]	March 2020	Segmentation techniques (SegNet, DRUNET) and ResNet classification model	Multiclass classification	3000 CT images of COVID_19 and pneumonia then testing on external data	-	94.33	91.22
[23]	June 2020	3D CNN model	Object detection and binary classification	618 CT images (219 images from 110 COVID-19 patients with mean age 50, 224 from IVAP patients with mean age 61, and 175 CT images from healthy people.	86.60	86.77	98.21
[42]	May 2020	U-net and ResNet32 models	Examine the effect of synthetic data on COVID-19 classification	2143 chest CTs related to 327 COVID-19-positive subjects across seven countries	90.06	-	-
[39]	March 2020	ML (RF and SVM) and CNN models	Utilizing CT images, patient symptoms for a binary classification task	626, negative cases 279 patients	83.77	81.8	84.2
[43]	June 2020	Multi-objective CNN model	Multiclass classification	312 CT scan images in addition to patient symptoms aggregated from COVID-19 patients in 9 days	93.40	91.00	89.00
[27]	August 2020	CNN based on ResNet 50 model	Binary classification	622 CT chest images from 122 for COVID-19 positive cases and 500 for normal cases	97.95	97.44	97.31
[44]	May 2020	DL model	Classification COVID-19 from pneumonia at early stages	219 images from 110 patients with COVID-19 (with mean age 50 years), 224 images from 224 patients with IAVP (mean age 61 years), and 175 images from 175 healthy cases (mean age 39 years)	86.72	86.5	86.5
[45]	June 2020	ImageNet and pre-trained model (ResNet50 and ResNet100) and CNN model	Binary classification	-	89.22	-	89.61
[46]	April 2020	Fully connected DL model	Binary classification	CT images from 1186 patients (132,583 CT slices). Data was divided into training, validation, and test datasets with percentage 7:2:1	96.21	95.0	96.21
[47]	May 2020	Using Generative Adversarial Networks and ResNet pretrained model to classify COVID-19 images	Binary classification	1- pneumonia dataset that includes (5863 X-ray images categorized: normal and pneumonia. 2- 624 images selected from normal and COVID-19 cases to demonstrate the effectiveness of the model	98.77	9.875	99.21

**Table 3:** Comparison between AI diagnosis algorithms based on X-ray for COVID-19 patients.

Ref.	Year	Method	Task	Dataset	Evaluation Measures		
					ACC (%)	P (%)	SN (%)
[52]	July 2020	Multi-image augmented Deep learning	Using both X-ray and CT images to provide binary classification model	100 cases of COVID-19 and non-COVID-19	99.4 for X-ray, 95.3 for CT scans	95.98	94.78
[53]	April 2020	VGG16, VGG19, ResNet, DenseNet, and InceptionV3	Evaluate the performance of CNN architecture and transfer learning in the COVID-19 classification process	1427 X-ray images include (224 COVID-19 + cases, 700 pf pneumonia, and 503 normal cases)	96.78	98.65	96.46
[54]	November 2020	Using SVM (Support Vector Machine), CNN (Conventional Neural Networks), ResNet50, InceptionResNetV2, Xception, VGGNet16	Examine the health status of the patient’s lung based on CT scan and X-ray	5857 Chest X-rays and 767 Chest CTs for COVID-19 positive cases	(84 for X-ray, 75 for CT scan)	-	-
[55]	September 2020	Machine learning techniques	Multiclass classification	350 images from confirmed cases, 220 images from suspected cases, and 130 images from normal cases	67.5	-	-
[56]	May 2020	Using encoder and decoder for segmentation, then use multilayer perceptron for image classification	Multitask model that includes three main steps: (1) image classification; (2) lesion segmentation; and (3) image reconstruction	1044 divided as (449 patients with COVID-19, 100 normal cases, 98 patients with lung cancer, and 398 with different pathology kinds	78	-	-
[57]	April 2020	COVID-net model: CNN model that trained first on ImageNet dataset then trained in COVIDx dataset	Analyzing patient data, predicting patient risk and hospitalization duration	13,975 images with many X-ray positive cases from various countries)	92.4	88.3	-
[50]	May 2020	Detecting features of X-ray image using CNN model then fed into SVM to make COVID-19 classification	Binary classification	Total of 50 images (25 for COVID-19 + 25 for pneumonia)	95.33	95.33	-
[58]	April 2020	COVID-Xnet model that builds on CNN models such as VGG19 and google MobileNet	Binary classification	Total of 50 images (25 for COVID-19 + 25 for non-COVID-19)	90		
[24]	May 2020	Using a darknet model for classification, YOLO for real-time object detection	Developed binary classification model that differentiates COVID-19 cases from healthy cases	1125 X-ray images (500 health cases, 125 COVID-19 positive cases, and 500 from pneumonia cases	98.02	95.13	95.3
[59]	October 2020	Deep learning and transfer learning models (ResNet50, inception V3, etc.)	COVID-19 diagnosis using X-ray images	100 X-ray images (50 COVID-19, 50 non-COVID-19) extracted form Dr. Chohen GitHub repository	98		
[60]	March 2020	Supervised pre-trained based 2D model called DeCOVNET	Diagnostic tool for COVID-19 detection using 3D images	499 CT images aggregated from 13 December 2019, to 23 January 2020, used for the training process. 131 CT images aggregated from 24 January to 6 February, were used for the testing process	90.01	90.65	91.21
[61]	February 2020	DL model based on relation extraction	Using 3D images to fast diagnose COVID-19 from pneumonia	CT scans images from 88 patients with positive COVID-19, 101 images from patients infected with bacteria pneumonia, and 86 images of healthy cases.	94.21	96.32	94.0
[62]	July 2020	Anomaly detection algorithm with efficient Net	Multiclass classification based on anomaly detection technology	Model firstly trained on 5977 images of viral pneumonia (no COVID-19) cases and 37,393 healthy cases. Then testing on the X-COVID dataset that include106 COVID-19 cases	72.77	71.30	-
[63]	June 2020	Using different pre-trained models (ResNet, AlexNet, SGDM- SqueezNet)	Using image augmentation in enhancing COVID-19 classification	423 X-rays of COVID-19 cases, 1485 X-rays of viral pneumonia cases, and 1579 of normal cases	98.2	96.7	98.2
[64]	June 2020	Feature optimization technique with Deep CNN model, known as COVXNet	COVID-19 detection	Viral, normal, and bacterial dataset available at (https://github.com/Perceptron21/CovXNet) (Last access date: 10 February 2021)	98.1	98.5	98.9
[65]	May 2020	Data augmentation and DL classification models	COVID-19 detection	A set of 5232 anterior–posterior (AP) images of children with ages from 1 to 5. It includes 1583 normal cases, 2780 bacterial pneumonia, and 1493 CXRs with COVID-19	99.25	-	-

**Table 4:** A comparison of ultrasound-based AI research for classifying COVID-19 patients.

Ref	Year	Method	Dataset	Task	Evaluation Measures		
					ACC	P	SN
[74]	April 2020	Machine learning	150 exams. Lung ultrasound was performed adopting the 12-region model, 6 on each side	Evaluating diagnostic accuracy of COVID-19 using lung ultrasound	82.1	-	-
[69]	May 2020	Deep learning	58,924 US frames	evaluate the applicability of ultrasound for making lung examination in COVID-19 patients	95	61	90
[67]	August 2020	Machine learning algorithms	1650 frames from 16 patients	Use lung US for 16 patients with COVID-19 to make the diagnosis	Positive predictive 86 and negative predictive 96	89	94
[70]	May 2020	VGG-16 pre-trained model followed by other hidden layers	2,392,963 frames form 64 videos	Provide automatic detection of COVID-19 based on US images	COVID-19: 97	96	79
					Pneumonia: 82	93	98
					Healthy: 63	0.01	1.00

**Table 5:** Distribution of cases and CFR of COVID-19 patients across various countries.

Country	Cases > 70 (%)	CFR	Death Age > 70 (%)
Canada	34.65	8.24	85.88
Italy	39.48	14.04	85.88
Denmark	17.01	4.71	87.45
Austria	16.82	3.85	85.12
Iceland	4.01	0.55	70.01
France	11.81	18.01	88.91
UK	16.62	16.14	82.33
USA	32.66	5.89	70.90
Spain	37.32	11.72	86.40
Sweden	21.01	7.44	88.94

**Table 6:** Correlation between COVID-19 and medical comorbidities.

Diseases	Correlation Percentage
Cardiovascular	14.08%
Diabetes	7.3%
Hypertension	7.0%
Respiratory diseases	12.4%
Liver disease	7.07%
Kidney failure diseases	11.32%

**Table 7:** Applications of using AI techniques in supporting COVID-19 patients.

Ref.	Application	Type of Data	AI Technique	Challenge
[203,204,205]	Chatbots to support COVID-19 patients and their relatives	Guidelines and information from a medical expert	NLP (i.e., information extraction, text summarization, and classification), speech recognition, and automated question answerers tools.	- Require a large amount of data to handle questions related to an unsaved query. - The challenge related to using various language expression (i.e., language slang)
[35,209,210]	Mining text to understand the community’s response towards governmental and health strategies (i.e., social distance, lockdown)	Text gathering from news, social media posts, healthcare, and governmental reports	NLP (i.e., information extraction, text summarization and classification)	- Privacy issues in different countries - Insufficient data may lead to skewed results. - Imprecise results leading to anxiety among the population.
[32,95,207]	Monitoring patients with temperature to maintain safety precautions) i.e., mask-wearing, social distancing, etc.)	Images extracted from infrared cameras in streets and public enterprises.	CNN models and pre-trained models (i.e., DesNet, AlexNet, etc.) and other computer vision tools and libraries	- Capturing the in-body temperature through remote sensors may lead to imprecise results. - Issues related to the invasion of privacy
[87,96,100,101,102]	Predict the spread of infection (number of expected patients, spread rate, disease peak, etc.)	Demographic data, population density, and compartmental tests,	Statistics tets and DL techniques (i.e., RNN and LSTM)	- Models such as compartmental models may be complex. - Insufficient data
[28,36,43,63,211,212,213,214,215,216,217,218,219]	COVID-19 medical diagnosis using medical images	Medical images (i.e., X-ray, CT scan, and ultrasound)	ML and DL CNN models, and AI computer vision tools	- Insufficient medical images lead to an imbalanced dataset.
[220,221,222,223,224]	Diagnosis and triage patient according to health status. Prescribe treatment, medical plan and make risk evaluation	Patient medical history (Electronic health record (EHR)), Patient symptoms, laboratory test result.	ML techniques (i.e., SVM, KNN, MLP, etc.), Fuzzy logic systems, and DL techniques (i.e., LSTM, RNN)	- Unavailability of patient’s data (therapeutic outcomes and physiological data). - Privacy issues - Incomplete data may lead to biased or accurate result in the prediction
[225,226,227]	Analyses of viral RNA and track genetic changes. Predict the viral structure of the second and third waves.	Protein sequence and viral RNA	DL and Deep reinforcement learning tools	- Analyzing a large dataset for RNA or protein sequence may take a long time, result in unexplainable models
[161,163,184,185,228,229,230,231]	Analyze chemical compounds and interaction for vaccine development	Viral structure, protein sequence, drug–drug interaction, drug–protein interaction, and protein–protein interaction.	DL models, computer vision tools, reinforcement learning, and optimization techniques	- Results need large bed experiments to be verified, which may take a long time. - Possibility of long-term risk.
[206,207,208]	Develop robots to support both patient and medical staff, cleaning, vital signs monitoring, deliver food and treatment	Training autonomous agent using environment simulation	DL models, computer vision tools, reinforcement learning, and optimization techniques	- Training autonomous agents and implementing them in machines may take great effort and time. - Maintaining a high level of safety must be guaranteed
[232]	Develop a reponse tracker (OXGRT) to capture the government policies and the degree of response	Aggregating huge dataset that is continuously updated	Use AI techniques to explore the empirical effect of government policies on the spread of COVID-19 cases	-

**Table 8:** Comparison between the COVID-19 medical images datasets.

Ref.	Type	Size	URL	Open-Source	Metadata
medseg.ai	CT scan	100 CT scans from 40 COVID-19 patients	http://medicalsegmentation.com/covid19/ (access date 20 February 2021)	Yes	Yes
[265]	CT scan	68,623 CT scan images for COVID-19 and non-COVID-19 images	-	No	No
[266]	CT scan	370 CT scan images for COVID-19 and non-COVID-19 images	-	Yes	No
[240]	X-ray	13,800 X-ray images for COVID-19 and phenomena	-	No	No
[236]	X-ray	100 X-ray images for COVID-19 and healthy class images	-	No	Yes
[241]	X-ray	230 X-ray images for COVID-19 and non-COVID-19 images	-	NO	No
[53]	X-ray	127 X-ray images for COVID-19 and non-COVID-19 images	-	No	No
[241]	X-ray	17,000 X-ray images for three class (COVID-19, healthy and phenomena	-	No	No
[242]	X-ray	2500 X-ray images for COVID-19 and non-COVID-19 images	-	Yes	NO
[243]	X-ray	4707 X-ray images for COVID-19 and non-COVID-19 images	-	Yes	Yes
Kaggle	X-ray	359 X-ray images for COVID-19 and non-COVID-19 patients	https://www.kaggle.com/bachrr/covid-chest-xray (access date 20 February 2021)	Yes	Yes
GitHub	X-ray	239 images for COVID-19-positive cases, in addition to some vital sings	https://github.com/agchung/Actualmed-COVID-chestxraydataset/tree/master/images, (access date 20 February 2021)	Yes	Yes
[25]	CT scan	34 CT scan images for COVID-19 and non-COVID-19 patients	https://github.com/UCSD-AI4H/COVID-CT, (access date 20 February 2021)	Yes	Yes
[70]	Ultrasound images	(654 COVID-19-positive subjects, 277 bacterial pneumonia, and 172 healthy subjects	https://github.com/jannisborn/covid19 pocus ultrasound/tree/master/data, (access date 20 February 2021)	Yes	Yes
[235]	CT scan and X-ray images	265 COVID-19 (165 X-ray, 100 CT scans)	https://github.com/ieee8023/covid-chestxray-dataset, (access date 20 February 2021)	Yes	Yes
EOR	CT scan and X-ray images	Various CT scan and X-ray images for COVID-19 patients	https://www.eurorad.org/advanced-search?search=COVID, (access date 20 February 2021)	No	Yes
BSTI	CT scan and X-ray images	Various CT scan and X-ray images for COVID-19 patients	https://bit.ly/BSTICovid19 Teaching Library (access date 20 February 2021)	No	Yes
[82]	Cough-sound	328 sound from 150 patient	-	No	No
[80]	Cough-sound	Cough and speech from 1079 normal and 92 COVID-19	https://coswara.iisc.ac.in (access date 20 February 2021)	Yes	Yes
[247]	Cough sound	Cough sound: 13 normal and 8 COVID-positive cases	https://coughtest.online (access date 20 February 2021)	Yes	Yes
GitHub	Cough sound	121 segmented coughs collected from 16 patient	https://github.com/virufy/covid (access date 20 February 2021)	Yes	Yes
[81]	Cough Sound	144 segmented coughs, aggregated from 28 patient	-	No	NO
[249]	Breathing sound	260 sound record aggregated from 52 COVID (32 male, 20 females) positive cases	-	No	Yes
[76]	Breathing sound	7000 unique samples, including 200 samples from COVID-19-confirmed cases	-	NO	Yes
[266]	Text data	Symptoms and health reports for 62 patients in South Korea	https://www.kaggle.com/kimjihoo/coronavirusdataset (access date 20 February 2021)	Yes	Yes
datahub	Text data	Time series symptoms from COVID-19 patients	https://datahub.io/core/covid-19 (access date 20 February 2021)	Yes	Yes
[69]	COVID-19 (Japan)	29 columns	https://www.kaggle.com/lisphilar/covid19-dataset-in-japan (access date 20 February 2021)	Yes	Yes
Word clouds	Covid-19 Text Dataset	Text data extracted from 13,202 scientific papers	https://github.com/Sarmentor/POS-Tagging-Wordcloud-with-R (access date 20 February 2021)	Yes	Yes
Kaggle	COVID-19 Predictors	28 demographic features about 96 countries (infection rate, number of ICU beds, death rate, etc)	https://www.kaggle.com/nightranger77/covid19-demographic-predictors (access date 20 February 2021)	Yes	Yes
Kaggle	COVID-19 country info	Include information about different countries, such as death rate, infection rate, and number of rapid tests	https://www.kaggle.com/koryto/countryinfo (access date 20 February 2021)	Yes	No
Kaggle	Coronavirus (COVID-19) Tweets	500,000 Tweets of users write the following hashtags: #coronavirus, #covid_19 #coronavirusoutbreak, #coronavirusPandemic, #covid19	https://www.kaggle.com/smid80/coronavirus-covid19-tweets (access date 20 February 2021)	Yes	Yes
[75]	COVID-19 Multilanguage Tweets Dataset	1200 M tweets collected using keywords related to COVID-19	https://sites.lafayette.edu/lopezbec/projects/covid-19-multilanguage-tweets-dataset/ (access date 20 February 2021)	Yes	Yes
[76]	COVID-19 Twitter Dataset	237 million tweets extracted from Twitter posts that mentioned “COVID” as a word or hashtag (e.g., COVID-19, COVID19)	https://dataverse.scholarsportal.info/dataset.xhtml?persistentId=doi:10.5683/SP2/PXF2CU (access date 20 February 2021)	yes	Yes
CDCP	Text data	Patient symptoms and report health status in	https://www.cdc.gov/coronavirus/2019-ncov/index.html https://www.coronavirus.gov/ (access date 20 February 2021)	Yes	Yes
NCBI	Genome data	Viral protein sequence	https://www.ncbi.nlm.nih.gov/genbank/sars-cov-2-seqs/ (access date 20 February 2021)	Yes	Yes
GISAID	Genome data	Viral protein sequence	https://www.gisaid.org/ (access date 20 February 2021)	Yes	Yes
GC	Genome data	Viral protein sequence	https://db.cngb.org/datamart/disease/DATAdis19/ (access date 20 February 2021)	Yes	Yes
EBI	Genome data	Viral structure, RNA, and protein sequence	https://www.covid19dataportal.org/ (access date 20 February 2021)	Yes	Yes
(NCBI).	Genome data	Viral protein sequence	https://registry.opendata.aws/ncbi-covid-19/ (access date 20 February 2021)	Yes	Yes
Zeng’s	Case reports	Reports on 20 projects, 16 report	http://open-source-covid-19.weileizeng.com/ (access date 20 February 2021)	Yes	Yes

BSTI: British Society of Thoracic Imaging; CDCP: Centers for Disease Control and Prevention in the US; GISAID: The GISAID organization; NCBI: NCBI GenBank; GC: GeneBank in China; EOR: European Organization for Radiology.

## Appendix A

**Table A1:** Distribution of gender, ages, and death rate among various countries. Note that these data were aggregated from online health organizations.

Country	Cases Date	Cases	Cases (% Male)	Cases (% Female)	Deaths Date	Deaths in Males	Deaths (% Male)	Deaths (% Female)	Death Date	Males Confirmed Percentage	Females Confirmed Percentage	Ratio between Males and Females (Males)
Afghanistan	12/15/2020	47,289	68.62%	31.38%	12/15/2020	1634	74.36%	25.64%	12/15/2020	3.74%	2.82%	1.33
Albania	01/02/2021	59,623	48%	52%	01/02/2021	1199	67%	33%	01/02/2021	2.81%	1.28%	2.2
Austria	01/06/2021	371,660	48.56%	51.44%	01/06/2021	6463	52.62%	47.38%	01/06/2021	1.88%	1.6%	1.18
Belgium	01/04/2021	649,570	44.58%	55.42%	01/04/2021	19724	49.05%	50.95%	01/04/2021	3.34%	2.79%	1.2
Bosnia and Herzegovina	01/03/2021	73,108	51.68%	48.32%	01/03/2021	2118	64.59%	35.41%	01/03/2021	3.62%	2.12%	1.71
Chile	12/31/2020	684,375	50.43%	49.57%	05/07/2020	294	60%	40%	05/07/2020	1.28%	0.97%	1.32
China	02/28/2020	55,924	51%	49%	02/28/2020	2114	64%	36%	02/28/2020	4.7%	2.8%	1.68
Costa Rica	01/03/2021	169,321	51.01%	48.99%	01/03/2021	2185	62.33%	37.67%	01/03/2021	1.58%	0.99%	1.59
Denmark	01/04/2021	170,787	48.92%	51.08%	01/04/2021	1226	55.79%	44.21%	01/04/2021	0.82%	0.62%	1.32
Ecuador	01/06/2021	217,377	52.65%	47.35%	12/13/2020	13874	66.51%	33.49%	12/13/2020	8.64%	4.87%	1.77
Equatorial Guinea	12/31/2020	4786	59.32%	40.68%	12/31/2020	86	70.93%	29.07%	12/31/2020	2.15%	1.28%	1.67
France	10/22/2020	1,047,083	47.46%	52.54%	12/24/2020	42853	58.66%	41.34%	10/20/2020	2.72%	1.7%	1.59
Germany	01/06/2021	1,793,732	47.38%	52.62%	01/06/2021	36470	52.22%	47.78%	01/06/2021	2.24%	1.85%	1.21
Haiti	12/31/2020	10127	57.2%	42.8%	12/31/2020	237	61.6%	38.4%	12/31/2020	2.52%	2.1%	1.2
Indonesia	01/05/2021	779,548	50%	50%	01/05/2021	23109	56.4%	43.6%	01/05/2021	3.34%	2.59%	1.29
Iran	03/17/2020	14,991	57%	43%	03/17/2020	853	59%	41%	03/17/2020	5.89%	5.43%	1.09
Israel	01/06/2021	461,644	50.97%	49.03%	01/06/2021	3527	57.36%	42.64%	01/06/2021	0.86%	0.66%	1.29
Italy	12/29/2020	2,049,915	48.48%	51.52%	12/29/2020	70799	56.9%	43.1%	12/29/2020	4.05%	2.89%	1.4
Jordan	01/04/2021	293,466	53%	47%	01/04/2021	3852	64.3%	35.7%	01/04/2021	1.59%	1%	1.6
Latvia	01/04/2021	43,118	42.86%	57.14%	01/04/2021	692	49%	51%	01/04/2021	1.83%	1.43%	1.28
Luxembourg	01/05/2021	47,149	50%	50%	01/05/2021	514	56%	44%	01/05/2021	1.22%	0.96%	1.27
Mexico	01/04/2021	1,454,974	50.4%	49.6%	01/04/2021	127533	63.41%	36.59%	01/04/2021	11.03%	6.47%	1.71
Morocco	07/18/2020	17,015	53%	47%	09/21/2020	1855	66.31%	33.69%	07/18/2020	2.98%	1.65%	1.8
Myanmar	09/10/2020	2265	53%	47%	09/28/2020	226	64.16%	35.84%	09/01/2020	1%	0.26%	3.84
Nepal	01/05/2021	262,784	65.11%	34.89%	12/23/2020	1795	69.86%	30.14%	12/23/2020	0.76%	0.61%	1.24
Nigeria	12/27/2020	73,043	61.85%	38.15%	11/15/2020	1218	75.29%	24.71%	11/15/2020	2.26%	1.28%	1.76
Northern Ireland	01/04/2021	81,222	46.08%	53.92%	01/06/2021	1383	51.19%	48.81%	01/06/2021	1.89%	1.54%	1.23
Portugal	01/03/2021	427,106	44.97%	55.03%	01/03/2021	7118	52.11%	47.89%	01/03/2021	1.93%	1.45%	1.33
Republic of Ireland	01/02/2021	101,791	47.67%	52.33%	01/02/2021	2263	51.22%	48.78%	01/02/2021	2.39%	2.07%	1.15
Romania	01/03/2021	643,559	45.98%	54.02%	01/03/2021	16057	59.7%	40.3%	01/03/2021	3.24%	1.86%	1.74
South Africa	01/05/2021	1,117,139	42.23%	57.77%	01/06/2021	27108	49.33%	50.67%	01/06/2021	2.83%	2.13%	1.33
South Korea	01/05/2021	64,979	48.91%	51.09%	01/05/2021	1007	50.35%	49.65%	01/05/2021	1.6%	1.51%	1.06
Spain	12/29/2020	1,888,148	46.98%	53.02%	05/21/2020	20518	57%	43%	05/21/2020	10.87%	6.3%	1.73
Sweden	01/06/2021	469,748	46.9%	53.1%	01/06/2021	8985	53.89%	46.11%	01/06/2021	2.2%	1.66%	1.32
Switzerland	01/06/2021	470,667	47.46%	52.54%	01/06/2021	7433	53.73%	46.27%	01/06/2021	1.79%	1.39%	1.29
Taiwan	01/05/2021	815	47.61%	52.39%	01/05/2021	7	85.71%	14.29%	01/05/2021	1.55%	0.23%	6.6
Thailand	11/01/2020	3784	56.37%	43.63%	11/01/2020	59	76.27%	23.73%	11/01/2020	2.11%	0.85%	2.49
Tunisia	10/20/2020	42,727	46%	54%	08/30/2020	77	68.75%	31.25%	08/30/2020	3.24%	1.29%	2.49
Turkey	10/25/2020	362,800	51%	49%	10/25/2020	9799	61.86%	38.14%	10/25/2020	3.28%	2.1%	1.56
Ukraine	01/05/2021	1,001,131	40.1%	59.9%	01/05/2021	17395	53.22%	46.78%	01/05/2021	2.31%	1.36%	1.7
USA	01/04/2021	15,091,901	47.71%	52.29%	12/26/2020	301671	54.21%	45.79%	10/27/2020	3.51%	2.76%	1.27
Wales	01/05/2021	161,233	45.23%	54.77%	01/05/2021	3738	56.5%	43.5%	01/05/2021	2.9%	1.84%	1.57

## References

[1] Fang S., Li K., Shen J., Liu S., Liu J., Yang L., Hu C.-D., Wan J. (2021). GESS: A database of global evaluation of SARS-CoV-2/hCoV-19 sequences. Nucleic Acids Res..

[2] Ludwig S., Zarbock A. (2020). Coronaviruses and SARS-CoV-2: A Brief Overview. Anesth. Analg..

[3] Bin S.Y., Heo J.Y., Song M.-S., Lee J., Kim E.-H., Park S.-J., Kwon H.-I., Kim S.M., Kim Y.-I., Si Y.-J. (2015). Environmental Contamination and Viral Shedding in MERS Patients during MERS-CoV Outbreak in South Korea. Clin. Infect. Dis..

[4] Wang L.-F., Shi Z., Zhang S., Field H., Daszak P., Eaton B.T. (2006). Review of Bats and SARS. Emerg. Infect. Dis..

[5] Chen B., Tian E.-K., He B., Tian L., Han R., Wang S., Xiang Q., Zhang S., El Arnaout T., Cheng W. (2020). Overview of lethal human coronaviruses. Signal Transduct. Target. Ther..

[6] Booth A.L., Abels E., McCaffrey P. (2021). Development of a prognostic model for mortality in COVID-19 infection using machine learning. Mod. Pathol..

[7] Sehla E., Eifan S., Hanif A. (2020). COVID-19 and Kingdom of Saudi Arabia. J. Clin. Microbiol. Biochem. Technol..

[8] Alharbi N.K., Alghnam S., Algaissi A., Albalawi H., Alenazi M.W., Albargawi A.M., Alharbi A.G., Alhazmi A., Al Qarni A., Alfarhan A. (2021). Nationwide Seroprevalence of SARS-CoV-2 in Saudi Arabia. J. Infect. Public Health.

[9] Orooji Y., Sohrabi H., Hemmat N., Oroojalian F. (2021). An Overview on SARS-CoV-2 (COVID-19) and Other Human Coronaviruses and Their Detection Capability via Amplification Assay, Chemical Sensing, Biosensing, Immunosensing, and Clinical Assays. NanoMicro Lett..

[10] Shereen M.A., Khan S., Kazmi A., Bashir N., Siddique R. (2020). COVID-19 infection: Emergence, transmission, and characteristics of human coronaviruses. J. Adv. Res..

[11] World Health Organization (WHO) (2020). Modes of Transmission of Virus Causing COVID-19: Implications for IPC Precaution Recommendations.

[12] Tong Z.-D., Tang A., Li K.-F., Li P., Wang H.-L., Yi J.-P., Zhang Y.-L., Yan J.-B. (2020). Potential presymptomatic transmission of SARS-CoV-2, Zhejiang Province, China, 2020. Emerg. Infect. Dis..

[13] To K.K.-W., Tsang O.T.-Y., Leung W.-S., Tam A.R., Wu T.-C., Lung D.C., Yip C.C.-Y., Cai J.-P., Chan J.M.-C., Chik T.S.-H. (2020). Temporal profiles of viral load in posterior oropharyngeal saliva samples and serum antibody responses during infection by SARS-CoV-2: An observational cohort study. Lancet Infect. Dis..

[14] Chan J.F.-W., Yuan S., Kok K.-H., To K.K.-W., Chu H., Yang J., Xing F., Liu J., Yip C.C.-Y., Poon R.W.-S. (2020). A familial cluster of pneumonia associated with the 2019 novel coronavirus indicating person-to-person transmission: A study of a family cluster. Lancet.

[15] Wu X., Wang Z., He Z., Li Y., Wu Y., Wang H., Liu Y., Hao F., Tian H. (2021). A follow-up study shows that recovered patients with re-positive PCR test in Wuhan may not be infectious. BMC Med..

[16] Armstrong S. (2020). Covid-19: Tests on students are highly inaccurate, early findings show. BMJ.

[17] Roy S. (2021). Physicians’ Dilemma of False-Positive RT-PCR for COVID-19: A Case Report. SN Compr. Clin. Med..

[18] Alserehi H.A., Alqunaibet A.M., Al-Tawfiq J.A., Alharbi N.K., Alshukairi A.N., Alanazi K.H., Bin Saleh G.M., Alshehri A.M., Almasoud A., Hashem A.M. (2021). Seroprevalence of SARS-CoV-2 (COVID-19) among healthcare workers in Saudi Arabia: Comparing case and control hospitals. Diagn. Microbiol. Infect. Dis..

[19] Singh V.K., Abdel-Nasser M., Pandey N., Puig D. (2021). LungINFseg: Segmenting COVID-19 Infected Regions in Lung CT Images Based on a Receptive-Field-Aware Deep Learning Framework. Diagnostics.

[20] Zietz M., Zucker J., Tatonetti N.P. (2020). Associations between blood type and COVID-19 infection, intubation, and death. Nat. Commun..

[21] Ahuja S., Panigrahi B.K., Dey N., Rajinikanth V., Gandhi T.K. (2021). Deep transfer learning-based automated detection of COVID-19 from lung CT scan slices. Appl. Intell..

[22] Jaiswal A., Gianchandani N., Singh D., Kumar V., Kaur M. (2020). Classification of the COVID-19 infected patients using DenseNet201 based deep transfer learning. J. Biomol. Struct. Dyn..

[23] López V., Čukić M. (2021). A dynamical model of SARS-CoV-2 based on people flow networks. Saf. Sci..

[24] Ozturk T., Talo M., Yildirim E.A., Baloglu U.B., Yildirim O., Acharya U.R. (2020). Automated detection of COVID-19 cases using deep neural networks with X-ray images. Comput. Biol. Med..

[25] Zhao J., He X., Yang X., Zhang Y., Zhang S., Xie P. (2020). COVID-CT-Dataset: A CT image dataset about COVID-19. arXiv.

[26] Ardakani A.A., Kanafi A.R., Acharya U.R., Khadem N., Mohammadi A. (2020). Application of deep learning technique to manage COVID-19 in routine clinical practice using CT images: Results of 10 convolutional neural networks. Comput. Biol. Med..

[27] Sotgiu G., Barassi A., Miozzo M., Saderi L., Piana A., Orfeo N., Colosio C., Felisati G., Davì M., Gerli A.G. (2020). SARS-CoV-2 specific serological pattern in healthcare workers of an Italian COVID-19 forefront hospital. BMC Pulm. Med..

[28] Sedik A., Iliyasu A., El-Rahiem B.A., Samea M.A., Abdel-Raheem A., Hammad M., Peng J., El-Samie F.A., El-Latif A. (2020). Deploying Machine and Deep Learning Models for Efficient Data-Augmented Detection of COVID-19 Infections. Viruses.

[29] Wang B., Jin S., Yan Q., Xu H., Luo C., Wei L., Zhao W., Hou X., Ma W., Xu Z. (2021). AI-assisted CT imaging analysis for COVID-19 screening: Building and deploying a medical AI system. Appl. Soft Comput..

[30] Wang S., Kang B., Ma J., Zeng X., Xiao M., Guo J., Cai M., Yang J., Li Y., Meng X. (2021). A deep learning algorithm using CT images to screen for Corona virus disease (COVID-19). Eur. Radiol..

[31] Jin C., Chen W., Cao Y., Xu Z., Tan Z., Zhang X., Deng L., Zheng C., Zhou J., Shi H. (2020). Development and evaluation of an artificial intelligence system for COVID-19 diagnosis. Nat. Commun..

[32] Malom Z., Rahman M.M.S., Nasrin S., Taha T.M., Asari V.K. (2020). COVID_MTNet: COVID-19 detection with multi-task deep learning approaches. arXiv.

[33] Sharma S. (2020). Drawing insights from COVID-19-infected patients using CT scan images and machine learning techniques: A study on 200 patients. Environ. Sci. Pollut. Res..

[34] Event E.P., Zhong A.L.I.U. (2020). Sciences Lung Infection Quantification of COVID-19 in CT Images with Deep Learning Author. arXiv.

[35] Nguyen T.T., Nguyen Q.V.H., Nguyen D.T., Hsu E.B., Yang S., Eklund P. (2020). Artificial Intelligence in the Battle against Coronavirus (COVID-19): A Survey and Future Research Directions. arXiv.

[36] Wang S., Zha Y., Li W., Wu Q., Li X., Niu M., Wang M., Qiu X., Li H., Yu H. (2020). A fully automatic deep learning system for COVID-19 diagnostic and prognostic analysis. Eur. Respir. J..

[37] Farid A.A., Selim G.I., Khater H.A.A. (2020). A Novel Approach of CT Images Feature Analysis and Prediction to Screen for Corona Virus Disease (COVID-19). Int. J. Sci. Eng. Res..

[38] Bernheim A., Mei X., Huang M., Yang Y., Fayad Z.A., Zhang N., Diao K., Lin B., Zhu X., Li K. (2020). Chest CT Findings in Coronavirus Disease-19 (COVID-19): Relationship to Duration of Infection. Radiology.

[39] Mei X., Lee H.-C., Diao K.-Y., Huang M., Lin B., Liu C., Xie Z., Ma Y., Robson P.M., Chung M. (2020). Artificial intelligence–enabled rapid diagnosis of patients with COVID-19. Nat. Med..

[40] Pu J., Leader J., Bandos A., Shi J., Du P., Yu J., Yang B., Ke S., Guo Y., Field J.B. (2020). Any unique image biomarkers associated with COVID-19?. Eur. Radiol..

[41] Zhang K., Liu X., Shen J., Li Z., Sang Y., Wu X., Zha Y., Liang W., Wang C., Wang K. (2020). Clinically Applicable AI System for Accurate Diagnosis, Quantitative Measurements, and Prognosis of COVID-19 Pneumonia Using Computed Tomography. Cell.

[42] Liu S., Georgescu B., Xu Z., Yoo Y., Chabin G., Chaganti S., Grbic S., Piat S., Teixeira B., Balachandran A. (2020). 3D Tomographic Pattern Synthesis for Enhancing the Quantification of COVID-19. arXiv.

[43] Singh D., Kumar V., Kaur M. (2020). Classification of COVID-19 patients from chest CT images using multi-objective differential evolution-based convolutional neural networks. Eur. J. Clin. Microbiol. Infect. Dis..

[44] Xu X., Jiang X., Ma C., Du P., Li X., Lv S., Yu L., Ni Q., Chen Y., Su J. (2020). A Deep Learning System to Screen Novel Coronavirus Disease 2019 Pneumonia. Engineering.

[45] Yang X., Chiu M.Y.L. (2020). Treatment of Adolescent Mental Disorders: Cross-Cultural Issues. The Encyclopedia of Child and Adolescent Development.

[46] Bai H.X., Wang R., Xiong Z., Hsieh B., Chang K., Halsey K., Tran T.M.L., Choi J.W., Wang D.-C., Shi L.-B. (2020). Artificial Intelligence Augmentation of Radiologist Performance in Distinguishing COVID-19 from Pneumonia of Other Origin at Chest CT. Radiology.

[47] Khalifa N.E.M., Taha M.H.N. (2020). Detection of Coronavirus (COVID-19) Associated Pneumonia based on Generative Adversarial Networks and a Fine-Tuned Deep Transfer Learning Model using Chest X-ray Dataset. arXiv.

[48] Wong H.Y.F., Lam H.Y.S., Fong A.H.-T., Leung S.T., Chin T.W.-Y., Lo C.S.Y., Lui M.M.-S., Lee J.C.Y., Chiu K.W.-H., Chung T.W.-H. (2020). Frequency and Distribution of Chest Radiographic Findings in Patients Positive for COVID-19. Radiology.

[49] Dansana D., Kumar R., Bhattacharjee A., Hemanth D.J., Gupta D., Khanna A., Castillo O. (2020). Early diagnosis of COVID-19-affected patients based on X-ray and computed tomography images using deep learning algorithm. Soft Comput..

[50] Sethy P.K., Behera S.K., Ratha P.K., Biswas P. (2020). Detection of coronavirus Disease (COVID-19) based on Deep Features and Support Vector Machine. Int. J. Math. Eng. Manag. Sci..

[51] Shi F., Xia L., Shan F., Song B., Wu D., Wei Y., Yuan H., Jiang H., He Y., Gao Y. (2021). Large-scale screening to distinguish between COVID-19 and community-acquired pneumonia using infection size-aware classification. Phys. Med. Biol..

[52] Purohit K., Kesarwani A., Kisku D.R., Dalui M. (2020). COVID-19 Detection on Chest X-Ray and CT Scan Images Using Multi-image Augmented Deep Learning Model. bioRxiv.

[53] Apostolopoulos I.D., Mpesiana T.A. (2020). Covid-19: Automatic detection from X-ray images utilizing transfer learning with convolutional neural networks. Phys. Eng. Sci. Med..

[54] Deng X., Shao H., Shi L., Wang X., Xie T. (2020). A classification–detection approach of COVID-19 based on chest X-ray and CT by using keras pretrained deep learning models. Comput. Model. Eng. Sci..

[55] Yasin R., Gouda W. (2020). Chest X-ray findings monitoring COVID-19 disease course and severity. Egypt. J. Radiol. Nucl. Med..

[56] Amyar A., Modzelewski R., Ruan S. (2020). Multi-Task Deep Learning Based CT Imaging Analysis for Covid-19: Classification and Segmentation General Electric Healthcare.

[57] Benmalek E., Elmhamdi J., Jilbab A. (2021). Comparing CT scan and chest X-ray imaging for COVID-19 diagnosis. Biomed. Eng. Adv..

[58] Hemdan E.E. (2003). COVIDX-Net: A Framework of Deep Learning Classifiers to Diagnose COVID-19 in X-Ray Images. arXiv.

[59] Minaee S., Kafieh R., Sonka M., Yazdani S., Soufi G.J. (2020). Deep-COVID: Predicting COVID-19 from chest X-ray images using deep transfer learning. Med. Image Anal..

[60] Zheng C., Deng X., Fu Q., Zhou Q. (2020). Deep Learning-based Detection for COVID-19 from Chest CT using Weak Label. MedRxiv.

[61] Song Y., Zheng S., Li L., Zhang X., Zhang X., Huang Z., Chen J., Wang R., Zhao H., Zha Y. (2021). Deep learning Enables Accurate Diagnosis of Novel Coronavirus (COVID-19) with CT images. IEEE/ACM Trans. Comput. Biol. Bioinform..

[62] Zhang J., Xie Y., Pang G., Liao Z., Verjans J., Li W., Sun Z., He J., Li Y., Shen C. (2021). Viral Pneumonia Screening on Chest X-Rays Using Confidence-Aware Anomaly Detection. IEEE Trans. Med. Imaging.

[63] Chowdhury M.E.H., Rahman T., Khandakar A., Mazhar R., Kadir M.A., Bin Mahbub Z., Islam K.R., Khan M.S., Iqbal A., Al Emadi N. (2020). Can AI Help in Screening Viral and COVID-19 Pneumonia?. IEEE Access.

[64] Mahmud T., Rahman A., Fattah S.A. (2020). CovXNet: A multi-dilation convolutional neural network for automatic COVID-19 and other pneumonia detection from chest X-ray images with transferable multi-receptive feature optimization. Comput. Biol. Med..

[65] Rajaraman S. (2020). Weakly Labeled Data Augmentation for Deep Learning: A Study on COVID-19 Detection in. Diagnostics.

[66] Wilhjelm B.J.E., Illum A., Kristensson M., Andersen O.T. (2016). Medical Diagnostic Ultrasound-Physical Principles and Imaging. http://bme.elektro.dtu.dk/jw/webbook/Ultrasound/main.pdf.

[67] Zhang Y.K., Li J., Yang J.P., Zhan Y., Chen J. (2015). Lung ultrasonography for the diagnosis of 11 patients with acute respiratory distress syndrome due to bird flu H7N9 infection. Virol. J..

[68] Convissar D.L., Gibson L.E., Berra L., Bittner E.A., Chang M.G. (2020). Application of Lung Ultrasound During the COVID-19 Pandemic: A Narrative Review. Anesthesia Analg..

[69] Roy S., Menapace W., Oei S., Luijten B., Fini E., Saltori C., Huijben I., Chennakeshava N., Mento F., Sentelli A. (2020). Deep Learning for Classification and Localization of COVID-19 Markers in Point-of-Care Lung Ultrasound. IEEE Trans. Med. Imaging.

[70] McDermott C., Łącki M., Sainsbury B., Henry J., Filippov M., Rossa C. (2021). Sonographic Diagnosis of COVID-19: A Review of Image Processing for Lung Ultrasound. Front. Big Data.

[71] Zhang Y., Xue H., Wang M., He N., Lv Z., Cui L. (2021). Lung Ultrasound Findings in Patients with Coronavirus Disease (COVID-19). Am. J. Roentgenol..

[72] Contreras-Ortiz S.H., Chiu T., Fox M.D. (2012). Ultrasound image enhancement: A review. Biomed. Signal Process. Control..

[73] Singh P., Mukundan R., De Ryke R. (2020). Feature Enhancement in Medical Ultrasound Videos Using Contrast-Limited Adaptive Histogram Equalization. J. Digit. Imaging.

[74] Sorlini C., Femia M., Nattino G., Bellone P., Gesu E., Francione P., Paternò M., Grillo P., Ruffino A., The Fenice Network (Italian Group for Clinical Research in Emergency Medicine) (2021). The role of lung ultrasound as a frontline diagnostic tool in the era of COVID-19 outbreak. Intern. Emerg. Med..

[75] Drosten C., Günther S., Preiser W., Van Der Werf S., Brodt H.R., Becker S., Rabenau H., Panning M., Kolesnikova L., Fouchier R.A.M. (2003). Identification of a Novel Coronavirus in Patients with Severe Acute Respiratory Syndrome. N. Engl. J. Med..

[76] Brown C., Chauhan J., Grammenos A., Han J., Hasthanasombat A., Spathis D., Xia T., Cicuta P., Mascolo C. Exploring Automatic Diagnosis of COVID-19 from Crowdsourced Respiratory Sound Data. Proceedings of the 26th ACM SIGKDD International Conference on Knowledge Discovery & Data Mining.

[77] Faezipour M., Abuzneid A. (2020). Smartphone-Based Self-Testing of COVID-19 Using Breathing Sounds. Telemed. e-Health.

[78] Lella K.K., Pja A. (2021). A literature review on COVID-19 disease diagnosis from respiratory sound data. AIMS Environ. Sci..

[79] World Health Organization (2020). World Health Organization and Others Report of the WHO-China Joint Mission on Coronavirus Disease 2019 (COVID-19).

[80] Sharma N., Krishnan P., Kumar R., Ramoji S., Chetupalli S.R., Ghosh P.K., Ganapathy S. (2020). Coswara—A Database of Breathing, Cough, and Voice Sounds for COVID-19 Diagnosis. arXiv.

[81] Lella K.K., Pja A. (2021). Automatic COVID-19 disease diagnosis using 1D convolutional neural network and augmentation with human respiratory sound based on parameters: Cough, breath, and voice. AIMS Public Health.

[82] Imran A., Posokhova I., Qureshi H.N., Masood U., Riaz M.S., Ali K., John C.N., Hussain I., Nabeel M. (2020). AI4COVID-19: AI enabled preliminary diagnosis for COVID-19 from cough samples via an app. Informatics Med. Unlocked.

[83] Bagad P., Dalmia A., Doshi J., Nagrani A., Bhamare P., Mahale A., Rane S., Agarwal N., Panicker R. (2020). Cough Against COVID: Evidence of COVID-19 Signature in Cough Sounds. arXiv.

[84] Han J., Qian K., Song M., Yang Z., Ren Z., Liu S., Liu J., Zheng H., Ji W., Koike T. (2020). An Early Study on Intelligent Analysis of Speech Under COVID-19: Severity, Sleep Quality, Fatigue, and Anxiety. arXiv.

[85] Wang N., Fu Y., Zhang H., Shi H. (2020). An evaluation of mathematical models for the outbreak of COVID-19. Precis. Clin. Med..

[86] Chae S.Y., Lee K., Lee H.M., Jung N., Le Q.A., Mafwele B.J., Lee T.H., Kim D.H., Lee J.W. (2020). Estimation of Infection Rate and Predictions of Disease Spreading Based on Initial Individuals Infected With COVID-19. Front. Phys..

[87] World Health Organization (2020). The First Few X Cases and Contacts (FFX) Investigation Protocol for Coronavirus Disease 2019 (COVID-19).

[88] Marmarelis V.Z. (2020). Predictive Modeling of Covid-19 Data in the US: Adaptive Phase-Space Approach. IEEE Open J. Eng. Med. Biol..

[89] Roda W.C., Varughese M.B., Han D., Li M.Y. (2020). Why is it difficult to accurately predict the COVID-19 epidemic?. Infect. Dis. Model..

[90] Liu M., Thomadsen R., Yao S. (2020). Forecasting the spread of COVID-19 under different reopening strategies. Sci. Rep..

[91] Li M.-T., Sun G.-Q., Zhang J., Zhao Y., Pei X., Li L., Wang Y., Zhang W.-Y., Zhang Z.-K., Jin Z. (2020). Analysis of COVID-19 transmission in Shanxi Province with discrete time imported cases. Math. Biosci. Eng..

[92] Ives A.R., Bozzuto C. (2021). Estimating and explaining the spread of COVID-19 at the county level in the USA. Commun. Biol..

[93] Kuniya T. (2020). Prediction of the Epidemic Peak of Coronavirus Disease in Japan, 2020. J. Clin. Med..

[94] Chen T.-M., Rui J., Wang Q.-P., Zhao Z.-Y., Cui J.-A., Yin L. (2020). A mathematical model for simulating the phase-based transmissibility of a novel coronavirus. Infect. Dis. Poverty.

[95] Ibrahim M., Al-Najafi A. (2020). Modeling, Control, and Prediction of the Spread of COVID-19 Using Compartmental, Logistic, and Gauss Models: A Case Study in Iraq and Egypt. Processes.

[96] Boldog P., Tekeli T., Vizi Z., Dénes A., Bartha F.A., Röst G. (2020). Risk Assessment of Novel Coronavirus COVID-19 Outbreaks Outside China. J. Clin. Med..

[97] Hilton J., Keeling M.J. (2020). Estimation of country-level basic reproductive ratios for novel Coronavirus (SARS-CoV-2/COVID-19) using synthetic contact matrices. PLoS Comput. Biol..

[98] Nishiura H., Chowell G. (2009). The Effective Reproduction Number as a Prelude to Statistical Estimation of Time-Dependent Epidemic Trends. Mathematical and Statistical Estimation Approaches in Epidemiology.

[99] Delamater P.L., Street E.J., Leslie T.F., Yang Y.T., Jacobsen K. (2019). Complexity of the Basic Reproduction Number (R0). Emerg. Infect. Dis..

[100] Musa S.S., Zhao S., Wang M.H., Habib A.G., Mustapha U.T., He D. (2020). Estimation of exponential growth rate and basic reproduction number of the coronavirus disease 2019 (COVID-19) in Africa. Infect. Dis. Poverty.

[101] Zhao S., Han L., He D., Qin J. (2020). Public awareness, news promptness and the measles outbreak in Hong Kong from March to April 2019. Infect. Dis..

[102] Nasab S.R., Zahiri A.-P., Roohi E. (2020). Prediction of peak and termination of novel coronavirus COVID-19 epidemic in Iran. Int. J. Mod. Phys. C.

[103] Lee S.Y., Lei B., Mallick B. (2020). Estimation of COVID-19 spread curves integrating global data and borrowing information. PLoS ONE.

[104] Tosepu R., Gunawan J., Effendy D.S., Ahmad L.O.A.I., Lestari H., Bahar H., Asfian P. (2020). Correlation between weather and Covid-19 pandemic in Jakarta, Indonesia. Sci. Total Environ..

[105] Almeshal A.M., Almazrouee A.I., Alenizi M.R., Alhajeri S.N. (2020). Forecasting the Spread of COVID-19 in Kuwait Using Compartmental and Logistic Regression Models. Appl. Sci..

[106] Sarhan A.R., Flaih M.H., Hussein T.A., Hussein K.R. (2020). Novel coronavirus (COVID-19) Outbreak in Iraq: The First Wave and Future Scenario. medRxiv.

[107] Tahir F.R. (2020). Epidemiological Characteristics of COVID-19 Ongoing Epidemic in Iraq.

[108] Saba A.I., Elsheikh A.H. (2020). Forecasting the prevalence of COVID-19 outbreak in Egypt using nonlinear autoregressive artificial neural networks. Process. Saf. Environ. Prot..

[109] Punn N.S., Sonbhadra S.K., Agarwal S. (2020). COVID-19 Epidemic Analysis using Machine Learning and Deep Learning. Algorithms.

[110] Wang Y., Hu M., Zhou Y., Li Q., Yao N., Zhai G., Zhang X.-P., Yang X. (2020). Unobtrusive and Automatic Classification of Multiple People’s Abnormal Respiratory Patterns in Real Time Using Deep Neural Network and Depth Camera. IEEE Internet Things J..

[111] Ye Y., Hou S., Fan Y., Zhang Y., Qian Y., Sun S., Peng Q., Ju M., Song W., Loparo K. (2020). α-Satellite: An AI-Driven System and Benchmark Datasets for Dynamic COVID-19 Risk Assessment in the United States. IEEE J. Biomed. Health Informatics.

[112] Rentsch C.T., Kidwai-Khan F., Tate J.P., Park L.S., Jr J.T.K., Skanderson M., Hauser R.G., Schultze A., Jarvis C.I., Holodniy M. (2020). Patterns of COVID-19 testing and mortality by race and ethnicity among United States veterans: A nationwide cohort study. PLoS Med..

[113] Chimmula V.K.R., Zhang L. (2020). Time series forecasting of COVID-19 transmission in Canada using LSTM networks. Chaos Solitons Fractals.

[114] Dutta S., Bandyopadhyay K.S. (2020). Machine Learning Approach for Confirmation of COVID-19 Cases: Positive, Negative, Death and Release. medRxiv.

[115] Latz C.A., Decarlo C., Boitano L., Png C.Y.M., Patell R., Conrad M.F., Eagleton M., Dua A. (2020). Blood type and outcomes in patients with COVID-19. Ann. Hematol..

[116] Zhao J., Yang Y., Huang H., Li D., Gu D., Lu X., Zhang Z., Liu L., Liu T., Liu Y. (2020). Relationship Between the ABO Blood Group and the Coronavirus Disease 2019 (COVID-19) Susceptibility. Clin. Infect. Dis..

[117] Brawley R.L. (1989). Clinical infectious diseases. Am. J. Infect. Control..

[118] Rubin R. (2020). Investigating Whether Blood Type Is Linked to COVID-19 Risk. JAMA.

[119] Algunmeeyn A., El-Dahiyat F., Altakhineh M.M., Azab M., Babar Z.-U.-D. (2020). Understanding the factors influencing healthcare providers’ burnout during the outbreak of COVID-19 in Jordanian hospitals. J. Pharm. Policy Pract..

[120] Abdelhafiz A.S., Ali A., Ziady H.H., Maaly A.M., Alorabi M., Sultan E.A. (2020). Prevalence, Associated Factors, and Consequences of Burnout Among Egyptian Physicians During COVID-19 Pandemic. Front. Public Health.

[121] Bekele F., Sheleme T., Fekadu G., Bekele K. (2020). Patterns and associated factors of COVID-19 knowledge, attitude, and practice among general population and health care workers: A systematic review. SAGE Open Med..

[122] Lindesmith L.C., Moe C.L., Marionneau S., Ruvoen N., Jiang X., Lindblad L., Stewart P.W., LePendu J., Baric R.S. (2003). Human susceptibility and resistance to Norwalk virus infection. Nat. Med..

[123] Chandekar S.A., Amonkar G.P., Desai H.M., Valvi N., Puranik G.V. (2017). Seroprevalence of transfusion transmitted in-fections in healthy blood donors: A 5-year tertiary care hospital experience. J. Lab. Physicians.

[124] Anderson J.L., May H.T., Knight S., Bair T.L., Muhlestein J.B., Knowlton K.U., Horne B.D. (2021). Association of Sociodemographic Factors and Blood Group Type With Risk of COVID-19 in a US Population. JAMA Netw. Open..

[125] Göker H., Aladağ-Karakulak E., Demiroğlu H., Ayaz C.M., Büyükaşik Y., İnkaya A.C., Aksu S., Sayinalp N., İbrahim, Haznedaroğlu C. (2020). The effects of blood group types on the risk of COVID-19 infection and its clinical outcome. Turkish J. Med. Sci..

[126] Zhang Y., Garner R., Salehi S., La Rocca M., Duncan D. (2021). Association between ABO blood types and coronavirus disease 2019 (COVID-19), genetic associations, and underlying molecular mechanisms: A literature review of 23 studies. Ann. Hematol..

[127] Williamson E.J., Walker A.J., Bhaskaran K., Bacon S., Bates C., Morton C.E., Curtis H.J., Mehrkar A., Evans D., Inglesby P. (2020). Factors associated with COVID-19-related death using Open SAFELY. Nature.

[128] Hoffmann C., Wolf E. (2021). Older age groups and country-specific case fatality rates of COVID-19 in Europe, USA and Canada. Infection.

[129] Sudharsanan N., Didzun O., Bärnighausen T., Geldsetzer P. (2020). The Contribution of the Age Distribution of Cases to COVID-19 Case Fatality Across Countries: A Nine-Country Demographic Study. Ann. Intern. Med..

[130] Omori R., Matsuyama R., Nakata Y. (2020). The age distribution of mortality from novel coronavirus disease (COVID-19) suggests no large difference of susceptibility by age. Sci. Rep..

[131] Ahmad S. (2020). Potential of age distribution profiles for the prediction of COVID-19 infection origin in a patient group. Inform. Med. Unlocked.

[132] Tadiri C.P., Gisinger T., Kautzky-Willer A., Kublickiene K., Herrero M.T., Raparelli V., Pilote L., Norris C.M. (2020). The influence of sex and gender domains on COVID-19 cases and mortality. Can. Med. Assoc. J..

[133] Stoian A.P., Pricop-Jeckstadt M., Pana A., Ileanu B.-V., Schitea R., Geanta M., Catrinoiu D., Suceveanu A.I., Serafinceanu C., Pituru S. (2020). Death by SARS-CoV 2: A Romanian COVID-19 multi-centre comorbidity study. Sci. Rep..

[134] (2020). WHO Gender and COVID-19.

[135] Lim S., Bae J.H., Kwon H.-S., Nauck M.A. (2021). COVID-19 and diabetes mellitus: From pathophysiology to clinical management. Nat. Rev. Endocrinol..

[136] Röst G., Bartha F.A., Bogya N., Boldog P., Dénes A., Ferenci T., Horváth K.J., Juhász A., Nagy C., Tekeli T. (2020). Early Phase of the COVID-19 Outbreak in Hungary and Post-Lockdown Scenarios. Viruses.

[137] Docherty A.B., Harrison E.M., Green C.A., Hardwick H.E., Pius R., Norman L., Holden K.A., Read J.M., Dondelinger F., Carson G. (2020). Features of 20 133 UK patients in hospital with covid-19 using the ISARIC WHO Clinical Characterisation Protocol: Prospective observational cohort study. BMJ.

[138] Tartof S.Y., Qian L., Hong M.V., Wei M.R., Nadjafi R.F., Fischer H., Li M.Z., Shaw D.S.F., Caparosa M.S.L., Nau C.L. (2020). Obesity and Mortality Among Patients Diagnosed With COVID-19: Results from an Integrated Health Care Organization. Ann. Intern. Med..

[139] Halpern B., Louzada M.L.D.C., Aschner P., Gerchman F., Brajkovich I., Faria-Neto J.R., Polanco F.E., Montero J., Juliá S.M.M., Lotufo P.A. (2021). Obesity and COVID-19 in Latin America: A tragedy of two pandemics—Official document of the Latin American Federation of Obesity Societies. Obes. Rev..

[140] Van Zyl-Smit R.N., Richards G., Leone F.T. (2020). Tobacco smoking and COVID-19 infection. Lancet Respir. Med..

[141] Malasinghe L.P., Ramzan N., Dahal K. (2019). Remote patient monitoring: A comprehensive study. J. Ambient. Intell. Humaniz. Comput..

[142] Aydin N., Yurdakul G. (2020). Assessing countries’ performances against COVID-19 via WSIDEA and machine learning algorithms. Appl. Soft Comput..

[143] Gülsen A., Yigitbas B.A., Uslu B., Drömann D., Kilinc O. (2020). The Effect of Smoking on COVID-19 Symptom Severity: Systematic Review and Meta-Analysis. Pulm. Med..

[144] Wang P., Sha J., Meng M., Wang C., Yao Q., Zhang Z., Sun W., Wang X., Qie G., Bai X. (2020). Risk factors for severe COVID-19 in middle-aged patients without comorbidities: A multicentre retrospective study. J. Transl. Med..

[145] Zhao W., Zhang J., Meadows M.E., Liu Y., Hua T., Fu B. (2020). A systematic approach is needed to contain COVID-19 globally. Sci. Bull..

[146] Murthy S., Gomersall C.D., Fowler R.A. (2020). Care for Critically Ill Patients With COVID-19. JAMA.

[147] Khan I.U., Aslam N., Aljabri M., Aljameel S.S., Kamaleldin M.M.A., Alshamrani F.M., Chrouf S.M.B. (2021). Computational Intelligence-Based Model for Mortality Rate Prediction in COVID-19 Patients. Int. J. Environ. Res. Public Health.

[148] Van Der Schaar M., Alaa A.M., Floto A., Gimson A., Scholtes S., Wood A., McKinney E., Jarrett D., Lio P., Ercole A. (2021). How artificial intelligence and machine learning can help healthcare systems respond to COVID-19. Mach. Learn..

[149] Liang W., Yao J., Chen A., Lv Q., Zanin M., Liu J., Wong S., Li Y., Lu J., Liang H. (2020). Early triage of critically ill COVID-19 patients using deep learning. Nat. Commun..

[150] Zhu J.S., Ge P., Jiang C., Zhang Y., Li X., Zhao Z., Zhang L., Duong T.Q. (2020). Deep-learning artificial intelligence analysis of clinical variables predicts mortality in COVID-19 patients. J. Am. Coll. Emerg. Physicians Open.

[151] Richardson S., Hirsch J.S., Narasimhan M., Crawford J.M., McGinn T., Davidson K.W., Barnaby D.P., Becker L.B., Chelico J.D., Cohen S.L. (2020). Presenting characteristics, comorbidities, and outcomes among 5700 patients hospitalized with COVID-19 in the New York City Area. JAMA.

[152] Thapa K., Mph B., Badal S., Bajgain B.B., Santana M.J. (2021). American Journal of Infection Control Prevalence of comorbidities among individuals with COVID-19: A rapid review of current literature. AJIC Am. J. Infect. Control.

[153] Luo L., Fu M., Li Y., Hu S., Luo J., Chen Z., Yu J., Li W., Dong R., Yang Y. (2020). The potential association between common comorbidities and severity and mortality of coronavirus disease 2019: A pooled analysis. Clin. Cardiol..

[154] Cavallaro M., Moiz H., Keeling M.J., Mccarthy N.D. (2020). Contrasting factors associated with COVID-19-related ICU and death outcomes: Interpretable multivariable analyses of the UK CHESS dataset. medRxiv.

[155] Aabed K., Lashin M.M.A. (2021). An analytical study of the factors that influence COVID-19 spread. Saudi J. Biol. Sci..

[156] Nakada L.Y.K., Urban R.C. (2020). COVID-19 pandemic: Environmental and social factors influencing the spread of SARS-CoV-2 in São Paulo, Brazil. Environ. Sci. Pollut. Res..

[157] Azuma K., Yanagi U., Kagi N., Kim H., Ogata M., Hayashi M. (2020). Environmental factors involved in SARS- CoV-2 transmission: Effect and role of indoor environmental quality in the strategy for COVID-19 infection control. Environ. Health Prev. Med..

[158] Bellantuono L., Monaco A., Tangaro S., Amoroso N., Aquaro V., Bellotti R. (2020). An equity-oriented rethink of global rankings with complex networks mapping development. Sci. Rep..

[159] Ivanov J., Polshakov D., Kato-Weinstein J., Zhou Q., Li Y., Granet R., Garner L., Deng Y., Liu C., Albaiu D. (2020). Quantitative Structure–Activity Relationship Machine Learning Models and their Applications for Identifying Viral 3CLpro- and RdRp-Targeting Compounds as Potential Therapeutics for COVID-19 and Related Viral Infections. ACS Omega.

[160] Zhou Y., Wang F., Tang J., Nussinov R., Cheng F. (2020). Articial intelligence in COVID-19 drug repurposing. Lancet Digit Health.

[161] Jain S., Potschka H., Chandra P.P., Tripathi M., Vohora D. (2021). Management of COVID-19 in patients with seizures: Mechanisms of action of potential COVID-19 drug treatments and consideration for potential drug-drug interactions with anti-seizure medications. Epilepsy Res..

[162] Mohanty S., Rashid M.H.A., Mridul M., Mohanty C., Swayamsiddha S. (2020). Application of Artificial Intelligence in COVID-19 drug repurposing. Diabetes Metab. Syndr. Clin. Res. Rev..

[163] Bung N., Krishnan S.R., Bulusu G., Roy A. (2021). De novo design of new chemical entities for SARS-CoV-2 using artificial intelligence. Future Med. Chem..

[164] Zhou Y., Hou Y., Shen J., Huang Y., Martin W., Cheng F. (2020). Network-based drug repurposing for novel coronavirus 2019-nCoV/SARS-CoV-2. Cell Discov..

[165] Avchaciov K., Burmistrova O., Fedichev P. (2020). AI for the Repurposing of Approved or Investigational Drugs against COVID-19. https://www.researchgate.net/publication/339998830_AI_for_the_repurposing_of_approved_or_investigational_drugs_against_COVID-19?channel=doi&linkId=5e71c42d299bf1571845af01&showFulltext=true.

[166] Wu Z., Pan S., Chen F., Long G., Zhang C., Yu P.S. (2021). A Comprehensive Survey on Graph Neural Networks. IEEE Trans. Neural Netw. Learn. Syst..

[167] Zhao S., Qin B., Liu T., Wang F. (2020). Biomedical Knowledge Graph Refinement with Embedding and Logic Rules. arXiv.

[168] Nicholson D.N., Greene C.S. (2020). Constructing knowledge graphs and their biomedical applications. Comput. Struct. Biotechnol. J..

[169] Richardson P., Griffin I., Tucker C., Smith D., Oechsle O., Phelan A., Rawling M., Savory E., Stebbing J. (2020). Baricitinib as potential treatment for 2019-nCoV acute respiratory disease. Lancet.

[170] Segler M.H.S., Preuss M., Waller M.P. (2018). Planning chemical syntheses with deep neural networks and symbolic AI. Nat. Cell Biol..

[171] Fauqueur J., Thillaisundaram A., Togia T. (2019). Constructing large scale biomedical knowledge bases from scratch with rapid annotation of interpretable patterns. arXiv.

[172] Ge Y., Tian T., Huang S., Wan F., Li J., Li S., Yang H., Hong L., Wu N., Yuan E. (2020). A data-driven drug repositioning framework discovered a potential therapeutic agent targeting COVID-19. bioRxiv.

[173] Loucera C., Esteban-Medina M., Rian K., Falco M.M., Dopazo J., Peña-Chilet M. (2020). Drug repurposing for COVID-19 using machine learning and mechanistic models of signal transduction circuits related to SARS-CoV-2 infection. Signal Transduct. Target. Ther..

[174] Hsieh K., Wang Y., Chen L., Zhao Z., Savitz S., Jiang X., Tang J., Kim Y. (2020). Drug Repurposing for COVID-19 using Graph Neural Network with Genetic, Mechanistic, and Epidemiological Validation 2020. arXiv.

[175] Zeng X., Song X., Ma T., Pan X., Zhou Y., Hou Y., Zhang Z., Li K., Karypis G., Cheng F. (2020). Repurpose Open Data to Discover Therapeutics for COVID-19 Using Deep Learning. J. Proteome Res..

[176] Liu X., Yang Z., Sang S., Lin H., Wang J., Xu B. (2019). Detection of protein complexes from multiple protein interaction networks using graph embedding. Artif. Intell. Med..

[177] Zhang H., Saravanan K.M., Yang Y., Hossain T., Li J., Ren X., Pan Y., Wei Y. (2020). Deep Learning Based Drug Screening for Novel Coronavirus 2019-nCov. Interdiscip. Sci. Comput. Life Sci..

[178] Wang R., Fang X., Lu Y., Wang S. (2004). The PDB bind database: Collection of binding affinities for protein-ligand complexes with known three-dimensional structures. J. Med. Chem..

[179] Gao K., Nguyen D.D., Wang R., Wei G. (2020). Machine intelligence design of 2019-nCoV drugs. bioRxiv.

[180] Forest R. (2018). Artificial Intelligence for Drug Discovery, Biomarker Development, and Generation of Novel Chemistry. Mol. Pharm..

[181] Batra R., Chan H., Kamath G., Ramprasad R., Cherukara M.J., Sankaranarayanan S.K. (2020). Screening of Therapeutic Agents for COVID-19 Using Machine Learning and Ensemble Docking Studies. J. Phys. Chem. Lett..

[182] Mall R., Elbasir A., al Meer H., Chawla S., Ullah E. (2020). Data-Driven Drug Repurposing for COVID-19. ChemRxiv.

[183] Jamshidi M.B., Lalbakhsh A., Talla J., Peroutka Z., Hadjilooei F., Lalbakhsh P., Jamshidi M., La Spada L., Mirmozafari M., Dehghani M. (2020). Artificial Intelligence and COVID-19: Deep Learning Approaches for Diagnosis and Treatment. IEEE Access.

[184] Tang B., He F., Liu D., Fang M., Wu Z., Xu D. (2020). AI-aided design of novel targeted covalent inhibitors against SARS-CoV-2. bioRxiv.

[185] Nguyen D.D., Gao K., Chen J., Wang R., Wei G.-W. (2020). Potentially highly potent drugs for 2019-nCoV. bioRxiv.

[186] Magar R., Yadav P., Farimani A.B. (2021). Potential neutralizing antibodies discovered for novel corona virus using machine learning. Sci. Rep..

[187] Krammer F. (2020). SARS-CoV-2 vaccines in development. Nature.

[188] Le T.T., Cramer J.P., Chen R., Mayhew S. (2020). Evolution of the COVID-19 vaccine development landscape. Nat. Rev. Drug Discov..

[189] Kumar A., Gupta P.K., Srivastava A. (2020). A review of modern technologies for tackling COVID-19 pandemic. Diabetes Metab. Syndr. Clin. Res. Rev..

[190] Fast E., Chen B. (2020). Potential T-cell and B-cell Epitopes of 2019-nCoV. bioRxiv.

[191] O’Connor P.J., Sperl-Hillen J., Fazio C.J., Averbeck B.M., Rank B.H., Margolis K. (2016). Outpatient diabetes clinical decision support: Current status and future directions. Diabet. Med..

[192] Parthasarathy R., Jaisoorya T.S., Thennarasu K., Murthy P. (2021). Mental health issues among health care workers during the COVID-19 pandemic—A study from India. Asian J. Psychiatry.

[193] Ong E., Wong M.U., Huffman A., He Y. (2020). COVID-19 Coronavirus Vaccine Design Using Reverse Vaccinology and Machine Learning. Front. Immunol..

[194] Bellantuono L., Marzano L., La Rocca M., Duncan D., Lombardi A., Maggipinto T., Monaco A., Tangaro S., Amoroso N., Bellotti R. (2021). Predicting brain age with complex networks: From adolescence to adulthood. NeuroImage.

[195] Bustin A., Fuin N., Botnar R.M., Prieto C. (2020). From Compressed-Sensing to Artificial Intelligence-Based Cardiac MRI Reconstruction. Front. Cardiovasc. Med..

[196] PGraffy P.M., Sandfort V., Summers R.M., Pickhardt P.J. (2019). Automated Liver Fat Quantification at Nonenhanced Abdominal CT for Population-based Steatosis Assessment. Radiology.

[197] Gozes O., Frid M., Greenspan H., Patrick D. (2020). Rapid AI Development Cycle for the Coronavirus (COVID-19) Pandemic: Initial Results for Automated Detection & Patient Monitoring using Deep Learning CT Image Analysis. arXiv.

[198] El-Rashidy N., El-Sappagh S., Islam S., El-Bakry H.M., Abdelrazek S. (2021). Mobile Health in Remote Patient Monitoring for Chronic Diseases: Principles, Trends, and Challenges. Diagnostics.

[199] Rahman A., Hossain M.S., Alrajeh N.A., Alsolami F. (2021). Adversarial Examples—Security Threats to COVID-19 Deep Learning Systems in Medical IoT Devices. IEEE Internet Things J..

[200] El-Rashidy N., El-Sappagh S., Abuhmed T., Abdelrazek S., El-Bakry H.M. (2020). Intensive Care Unit Mortality Prediction: An Improved Patient-Specific Stacking Ensemble Model. IEEE Access.

[201] El-Rashidy N., El-Sappagh S., Islam S.M.R., El-Bakry H.M., Abdelrazek S. (2020). End-To-End Deep Learning Framework for Coronavirus (COVID-19) Detection and Monitoring. Electronics.

[202] Yang G., Zhang H., Firmin D., Li S. (2021). Recent advances in artificial intelligence for cardiac imaging. Comput. Med. Imaging Graph..

[203] Gagneux-Brunon A., Detoc M., Bruel S., Tardy B., Rozaire O., Frappe P., Botelho-Nevers E. (2021). Intention to get vaccinations against COVID-19 in French healthcare workers during the first pandemic wave: A cross-sectional survey. J. Hosp. Infect..

[204] Alhasan M., Hasaneen M. (2021). Digital Imaging, Technologies and Artificial Intelligence Applications during COVID-19 pandemic. Comput. Med. Imaging Graph..

[205] Zeng Z., Chen P.-J., Lew A.A. (2020). From high-touch to high-tech: COVID-19 drives robotics adoption. Tour. Geogr..

[206] Estrada M.A.R., Ndoma A. (2019). The uses of unmanned aerial vehicles–UAV’s- (or drones) in social logistic: Natural disasters response and humanitarian relief aid. Procedia Comput. Sci..

[207] Tavakoli M., Carriere J., Torabi A. (2020). Robotics, Smart Wearable Technologies, and Autonomous Intelligent Systems for Healthcare During the COVID-19 Pandemic: An Analysis of the State of the Art and Future Vision. Adv. Intell. Syst..

[208] AAbd-Alrazaq A., Alhuwail D., Househ M., Hamdi M., Shah Z. (2020). Top Concerns of Tweeters During the COVID-19 Pandemic: Infoveillance Study. J. Med. Internet Res..

[209] Torales J., O’Higgins M., Castaldelli-Maia J.M., Ventriglio A. (2020). The outbreak of COVID-19 coronavirus and its impact on global mental health. Int. J. Soc. Psychiatry.

[210] Li S., Wang Y., Xue J., Zhao N., Zhu T. (2020). The Impact of COVID-19 Epidemic Declaration on Psychological Consequences: A Study on Active Weibo Users. Int. J. Environ. Res. Public Health.

[211] Jaiswal A.K., Tiwari P., Kumar S., Gupta D., Khanna A., Rodrigues J.J. (2019). Identifying pneumonia in chest X-rays: A deep learning approach. Measurement.

[212] Civit-Masot J., Luna-Perejón F., Morales M.D., Civit A. (2020). Deep Learning System for COVID-19 Diagnosis Aid Using X-ray Pulmonary Images. Appl. Sci..

[213] Apostolopoulos I.D., Aznaouridis S.I., Tzani M.A. (2020). Extracting Possibly Representative COVID-19 Biomarkers from X-ray Images with Deep Learning Approach and Image Data Related to Pulmonary Diseases. J. Med. Biol. Eng..

[214] Horry M.J., Chakraborty S., Paul M., Ulhaq A., Pradhan B., Saha M., Shukla N. (2020). COVID-19 Detection Through Transfer Learning Using Multimodal Imaging Data. IEEE Access.

[215] Pereira R.M., Bertolini D., Teixeira L.O., Silla C.N., Costa Y.M.G. (2020). Computer Methods and Programs in Biomedicine COVID-19 identification in chest X-ray images on flat and hierarchical classification scenarios. Comput. Methods Prog. Biomed..

[216] Yadav S., Kaur J., Pathak Y., Jadhav S. (2015). Chest X-ray Scanning Based Detection of COVID-19 Using Deep Convolutional Neural Network. https://assets.researchsquare.com/files/rs-58833/v1_stamped.pdf.

[217] Jain G., Mittal D., Thakur D., Mittal M.K. (2020). A deep learning approach to detect Covid-19 coronavirus with X-Ray images. Biocybern. Biomed. Eng..

[218] Yu C., Zhou M., Liu Y., Guo T., Ou C., Yang L., Li Y., Li D., Hu X., Shuai L. (2020). Characteristics of asymptomatic COVID-19 infection and progression: A multicenter, retrospective study. Virulence.

[219] Jiang X., Coffee M., Bari A., Wang J., Jiang X., Huang J., Shi J., Dai J., Cai J., Zhang T. (2020). Towards an Artificial Intelligence Framework for Data-Driven Prediction of Coronavirus Clinical Severity. Comput. Mater. Contin..

[220] Shelke A., Inamdar M., Shah V., Tiwari A., Hussain A., Chafekar T., Mehendale N. (2021). Chest X-ray Classification Using Deep Learning for Automated COVID-19 Screening. SN Comput. Sci..

[221] Soomro T.A., Zheng L., Afifi A.J., Ali A., Yin M., Gao J. (2021). Artificial intelligence (AI) for medical imaging to combat coronavirus disease (COVID-19): A detailed review with direction for future research. Artif. Intell. Rev..

[222] Rahmatizadeh S., Valizadeh-Haghi S., Dabbagh A. (2020). The role of artificial intelligence in management of critical COVID-19 patients. J. Cell. Mol. Anesth..

[223] Feng C., Wang L., Chen X., Zhai Y., Zhu F., Chen H., Wang Y., Su X., Huang S., Tian L. (2021). A novel artificial intelligence-assisted triage tool to aid in the diagnosis of suspected COVID-19 pneumonia cases in fever clinics. Ann. Transl. Med..

[224] Yan L., Zhang H.-T., Goncalves J., Xiao Y., Wang M., Guo Y., Sun C., Tang X., Jin L., Zhang M. (2020). A machine learning-based model for survival prediction in patients with severe COVID-19 infection. MedRxiv.

[225] Senior A.W., Evans R., Jumper J., Kirkpatrick J., Sifre L., Green T., Qin C., Žídek A., Nelson A.W.R., Bridgland A. (2020). Improved protein structure prediction using potentials from deep learning. Nature.

[226] Heo L., Feig M. (2020). Modeling of Severe Acute Respiratory Syndrome Coronavirus 2 (SARS-CoV-2) Proteins by Machine Learning and Physics-Based Refinement. bioRxiv.

[227] Nguyen T.T., Abdelrazek M., Nguyen D.T., Aryal S., Nguyen D.T., Khatami A. (2020). Origin of Novel Coronavirus (COVID-19): A Computational Biology Study using Artificial Intelligence. bioRxiv.

[228] Li X., Yu J., Zhang Z., Ren J., Peluffo A.E., Zhang W., Zhao Y., Wu J., Yan K., Cohen D. (2021). Network bioinformatics analysis provides insight into drug repurposing for COVID-19. Med. Drug Discov..

[229] Zhavoronkov A., Aladinskiy V., Zhebrak A., Zagribelnyy B., Terentiev V., Bezrukov D.S., Polykovskiy D., Shayakhmetov R., Filimonov A., Orekhov P. (2020). Potential 2019-nCoV 3C-like protease inhibitors designed using generative deep learning approaches. ChemRxiv.

[230] Nguyen D.D., Gao K., Wang M., Wei G.-W. (2019). MathDL: Mathematical deep learning for D3R Grand Challenge 4. J. Comput. Mol. Des..

[231] Robson B. (2020). Computers and viral diseases. Preliminary bioinformatics studies on the design of a synthetic vaccine and a pre-ventative peptidomimetic antagonist against the SARS-CoV-2 (2019-nCoV, COVID-19) coronavirus. Comput. Biol. Med..

[232] HealthMap (2020). Contagious Disease Surveillance. https://healthmap.org/en/.

[233] James A., Dasarathy B.V. (2017). A Review of Feature and Data Fusion with Medical Images. Matlab.

[234] Hu S., Gao Y., Niu Z., Jiang Y., Li L., Xiao X., Wang M., Fang E.F., Menpes-Smith W., Xia J. (2020). Weakly Supervised Deep Learning for COVID-19 Infection Detection and Classification from CT Images. IEEE Access.

[235] Li X., Zeng W., Li X., Chen H., Shi L., Li X., Xiang H., Cao Y., Chen H., Liu C. (2020). CT imaging changes of corona virus disease 2019(COVID-19): A multi-center study in Southwest China. J. Transl. Med..

[236] Cohen J.P., Morrison P., Dao L. (2020). COVID-19 Image Data Collection. arXiv.

[237] Cohen J.P., Morrison P., Dao L., Roth K., Duong T.Q., Ghassemi M. (2020). COVID-19 Image Data Collection: Prospective predictions are the future. arXiv.

[238] COVID-19 CT Lung and Infection Segmentation Dataset. https://zenodo.org/record/3757476#.X-9uTtj7Q2w.

[239] Yoo S.H., Geng H., Chiu T.L., Yu S.K., Cho D.C., Heo J., Choi M.S., Choi I.H., Van C.C., Nhung N.V. (2020). Deep Learning-Based Decision-Tree Classifier for COVID-19 Diagnosis from Chest X-ray Imaging. Front. Med..

[240] Wang L., Lin Z.Q., Wong A. (2020). COVID-Net: A tailored deep convolutional neural network design for detection of COVID-19 cases from chest X-ray images. Sci. Rep..

[241] Kermany D.S., Goldbaum M., Cai W., Valentim C.C., Liang H., Baxter S.L., McKeown A., Yang G., Wu X., Yan F. (2018). Identifying Medical Diagnoses and Treatable Diseases by Image-Based Deep Learning. Cell.

[242] Sharma A., Rani S., Gupta D. (2020). Artificial Intelligence-Based Classification of Chest X-Ray Images into COVID-19 and Other Infectious Diseases. Int. J. Biomed. Imaging.

[243] Shuja J., Alanazi E., Alasmary W., Alashaikh A. (2021). COVID-19 open source data sets: A comprehensive survey. Appl. Intell..

[244] Signoroni A., Savardi M., Benini S., Adami N., Leonardi R., Gibellini P., Vaccher F., Ravanelli M., Borghesi A., Maroldi R. (2021). BS-Net: Learning COVID-19 pneumonia severity on a large chest X-ray dataset. Med. Image Anal..

[245] Wong A., Lin Z.Q., Wang L., Chung A.G., Shen B., Abbasi A., Hoshmand-Kochi M., Duong T.Q. (2020). COVIDNet-S: Towards computer-aided severity assessment via training and validation of deep neural networks for geographic extent and opacity extent scoring of chest X-rays for SARS-CoV-2 lung disease severity. arXiv.

[246] Pia L. (2020). SARS-CoV-2-reactive T cells in patients and healthy donors. Nat. Rev. Immunol..

[247] Cohen-McFarlane M., Goubran R., Knoefel F. (2020). Novel Coronavirus Cough Database: NoCoCoDa. IEEE Access.

[248] Pahar M., Klopper M., Warren R., Niesler T. (2020). COVID-19 Cough Classification using Machine Learning and Global Smartphone Recordings. arXiv.

[249] Rahman M.M., Davis D.N. (2013). Addressing the Class Imbalance Problem in Medical Datasets. Int. J. Mach. Learn. Comput..

[250] Dash T., Mishra S., Panda G., Satapathy S. (2021). Detection of COVID-19 from speech signal using bio-inspired based cepstral features. Pattern Recognit..

[251] Dong E., Du H., Gardner L. (2020). An interactive web-based dashboard to track COVID-19 in real time. Lancet Infect. Dis..

[252] Xu B., Kraemer M.U.G., Gutierrez B., Mekaru S., Sewalk K., Loskill A., Wang L., Cohn E., Hill S., Zarebski A. (2020). Open access epidemiological data from the COVID-19 outbreak. Lancet Infect. Dis..

[253] Xu B., Gutierrez B., Mekaru S., Sewalk K., Goodwin L., Loskill A., Cohn E.L., Hswen Y., Hill S.C., Cobo M.M. (2020). Epidemiological data from the COVID-19 outbreak, real-time case information. Sci. Data.

[254] Kucharski A.J., Russell T.W., Diamond C., Liu Y., Edmunds J., Funk S., Eggo R.M., Sun F., Jit M., Munday J.D. (2020). Early dynamics of transmission and control of COVID-19: A mathematical modelling study. Lancet Infect. Dis..

[255] Wells C.R., Sah P., Moghadas S.M., Pandey A., Shoukat A., Wang Y., Wang Z., Meyers L.A., Singer B.H., Galvani A.P. (2020). Impact of international travel and border control measures on the global spread of the novel 2019 coronavirus outbreak. Proc. Natl. Acad. Sci. USA.

[256] Tian H., Liu Y., Li Y., Wu C.-H., Chen B., Kraemer M.U.G., Li B., Cai J., Xu B., Yang Q. (2020). An investigation of transmission control measures during the first 50 days of the COVID-19 epidemic in China. Science.

[257] Kraemer M.U.G., Yang C.-H., Gutierrez B., Wu C.-H., Klein B., Pigott D.M., Du Plessis L., Faria N.R., Li R., Hanage W.P. (2020). The effect of human mobility and control measures on the COVID-19 epidemic in China. Science.

[258] Anzai A., Kobayashi T., Linton N.M., Kinoshita R., Hayashi K., Suzuki A., Yang Y., Jung S.-M., Miyama T., Akhmetzhanov A.R. (2020). Assessing the Impact of Reduced Travel on Exportation Dynamics of Novel Coronavirus Infection (COVID-19). J. Clin. Med..

[259] Petherick A., Kira B., Cameron-Blake E., Tatlow H., Hallas L., Hale T., Phillips T., Zhang Y. (2020). Variation in Government Responses to COVID-19.

[260] Kleinberg B., van der Vegt I., Mozes M. (2020). bMeasuring Emotions in the COVID-19 Real World Worry Dataset. arXiv.

[261] Banda J.M., Tekumalla R., Wang G., Yu J., Liu T., Ding Y., Chowell G. (2020). A large-scale COVID-19 Twitter chatter dataset for open scientific research—An international collaboration. arXiv.

[262] Alqurashi S., Alhindi A., Alanazi E. (2020). Large Arabic Twitter Dataset on COVID-19. arXiv.

[263] Barbosa R.D., Fernandes M.A.C. (2020). Data stream dataset of SARS-CoV-2 genome. Data Br..

[264] Pickett B., Sadat E.L., Zhang Y., Noronha J.M., Squires R.B., Hunt V., Liu M., Kumar S., Zaremba S., Gu Z. (2011). ViPR: An open bioinformatics database and analysis resource for virology research. Nucleic Acids Res..

[265] Wu Y.-H., Gao S.-H., Mei J., Xu J., Fan D.-P., Zhang R.-G., Cheng M.-M. (2021). JCS: An Explainable COVID-19 Diagnosis System by Joint Classification and Segmentation. IEEE Trans. Image Process..

[266] Ma J., Ge C., Wang Y., An X., Gao J., Yu Z., Zhang M., Liu X., Deng X., Cao S. (2020). Covid-19 CT Lung and Infection Segmentation Dataset. https://zenodo.org/record/3757476#.YMqve6gzaUk.

[267] Kim J. DS4C Patient Policy Province Dataset: A Comprehensive COVID-19 Dataset for Causal and Epidemiological Analysis. Proceedings of the 4th Conference on Neural Information Processing Systems (NeurIPS 2020).

[268] Wynants L., Van Calster B., Collins G.S., Riley R.D., Heinze G., Schuit E., Bonten M.M.J., Dahly D.L., Damen J., Debray T.P. (2020). Prediction models for diagnosis and prognosis of covid-19: Systematic review and critical appraisal. BMJ.

[269] Asraf A., Islam Z., Haque R., Islam M. (2020). Deep Learning Applications to Combat Novel Coronavirus (COVID-19) Pandemic. SN Comput. Sci..

[270] Bhattacharya S., Maddikunta P.K.R., Pham Q.-V., Gadekallu T.R., Chowdhary C.L., Alazab M., Piran J. (2021). Deep learning and medical image processing for coronavirus (COVID-19) pandemic: A survey. Sustain. Cities Soc..

[271] Di Castelnuovo A., Bonaccio M., Costanzo S., Gialluisi A., Antinori A., Berselli N., Blandi L., Bruno R., Cauda R., Guaraldi G. (2020). Common cardiovascular risk factors and in-hospital mortality in 3,894 patients with COVID-19: Survival analysis and machine learning-based findings from the multicentre Italian CORIST Study. Nutr. Metab. Cardiovasc. Dis..

[272] Cabitza F., Campagner A., Ferrari D., Di Resta C., Ceriotti D., Sabetta E., Colombini A., De Vecchi E., Banfi G., Locatelli M. (2021). Development, evaluation, and validation of machine learning models for COVID-19 detection based on routine blood tests. Clin. Chem. Lab. Med..

[273] Xing C., Li Q., Du H., Kang W., Lian J., Yuan L. (2020). Lung ultrasound findings in patients with COVID-19 pneumonia. Crit. Care.

[274] Liao H., Balocco S., Wang G., Zhang F., Liu Y., Ding Z., Duong L., Phellan R., Zahnd G., Breininger K. Machine Learning and Medical Engineering for Cardiovascular Health and Intravascular Imaging and Computer Assisted Stenting. Proceedings of the CVII-STENT 2019, Held Conjunction with MICCAI 11794.

[275] Al-Antari M.A., Hua C.-H., Bang J., Lee S. (2021). Fast deep learning computer-aided diagnosis of COVID-19 based on digital chest x-ray images. Appl. Intell..

[276] Ismael A.M., Şengür A. (2021). Deep learning approaches for COVID-19 detection based on chest X-ray images. Expert Syst. Appl..

[277] Das N.N., Kumar N., Kaur M., Kumar V., Singh D. (2020). Automated Deep Transfer Learning-Based Approach for Detection of COVID-19 Infection in Chest X-rays. IRBM.

[278] Yeşilkanat C.M. (2020). Spatio-temporal estimation of the daily cases of COVID-19 in worldwide using random forest machine learning algorithm. Chaos Solitons Fractals.

[279] Trivedy S., Goyal M., Mohapatra P.R., Mukherjee A. (2020). Design and Development of Smartphone-Enabled Spirometer With a Disease Classification System Using Convolutional Neural Network. IEEE Trans. Instrum. Meas..

[280] Vaishya R., Javaid M., Khan I.H., Haleem A. (2020). Artificial Intelligence (AI) applications for COVID-19 pandemic. Diabetes Metab. Syndr. Clin. Res. Rev..

[281] Narin A., Kaya C., Pamuk Z. (2021). Automatic detection of coronavirus disease (COVID-19) using X-ray images and deep convolutional neural networks. Pattern Anal. Appl..

[282] Vaid S., Cakan C., Bhandari M. (2020). Using Machine Learning to Estimate Unobserved COVID-19 Infections in North America. J. Bone Jt. Surg. Am. Vol..

[283] Fahmy A.E., El-desouky M.M., Mohamed A.S.A. (2020). Epidemic Analysis of COVID-19 in Egypt, Qatar and Saudi Arabia using the Generalized SEIR Model. medRxiv.

[284] Khanday A.M.U.D., Rabani S.T., Khan Q.R., Rouf N., Din M.M.U. (2020). Machine learning based approaches for detecting COVID-19 using clinical text data. Int. J. Inf. Technol..

[285] Guo X., Mirzaalian H., Sabir E. (2020). CORD19STS: COVID-19 Semantic Textual Similarity Dataset. arXiv.

[286] Panwar H., Gupta P., Siddiqui M.K., Morales-Menendez R., Bhardwaj P., Singh V. (2020). A deep learning and grad-CAM based color visualization approach for fast detection of COVID-19 cases using chest X-ray and CT-Scan images. Chaos Solitons Fractals.

